# Supplementary material for: Towards reliable quantification of cell state velocities
Source: PLoS Comput Biol. 2022 Sep 28;18(9):e1010031. doi: 10.1371/journal.pcbi.1010031 (PMC9550177; doi:10.1371/journal.pcbi.1010031)
Supplement: S1 Table of contents — (PDF) [file pcbi.1010031.s001.pdf]

Table of contents for
"Towards reliable quantification of cell state velocities"

Valérie Marot-Lassauzaie[◑], Brigitte Joanne Bouman[◑], Fearghal Declan Donaghy, Yasmin Demerdash, Marieke Alida Gertruda Essers, Laleh Haghverdi*

◑These authors contributed equally to this work.
* laleh.haghverdi@mdc-berlin.de

Table of contents

Introduction

Methods

- Dynamical inference
  - The time scale over which average cell state velocities are reported
  - Scale invariance of gene-wise velocity components
  - First approach: κ-velo
  - Second approach: eco-velo
- Visualisation
  - Nyström projection (velocity visualisation for κ-velo)
  - Visualisation for eco-velo
- Processing
  - Processing pipeline of κ-velo
  - Processing pipeline of eco-velo
- Overview of the workflow for κ-velo and eco-velo
- Simulation data
- Real data

Results

- PCA and Nyström projection faithfully represent the high-dimensional velocity vectors
- κ-velo recovers simulated velocities
- Careful processing prevents introduction of artefacts
- κ-velo explains cell state plasticities and speed of transcriptional change in pancreas endocrinogenesis
- κ-velo recovers multiple differentiation paths in hematopoietic system
- Eco-velo approximates cell state velocities using minimal data processing and computation
- Computational efficiency of the methods
- Data and software availability

Discussion

Supporting information

References
