# Supplementary material for: Towards reliable quantification of cell state velocities
Source: PLoS Comput Biol. 2022 Sep 28;18(9):e1010031. doi: 10.1371/journal.pcbi.1010031 (PMC9550177; doi:10.1371/journal.pcbi.1010031)
Supplement: S1 Appendix — Details on theory, the algorithms, the simulation and processing of the data. (PDF) [file pcbi.1010031.s002.pdf]

Supplementary information for
"Towards reliable quantification of cell state velocities"

Valérie Marot-Lassauzaie◕, Brigitte Joanne Bouman◕, Fearghal Declan Donaghy, Yasmin Demerdash, Marieke Alida Gertruda Essers, Laleh Haghverdi*

◕These authors contributed equally to this work.
* laleh.haghverdi@mdc-berlin.de

Contents

Note A: Scaling of unspliced counts 1
Note B: Expectation maximisation 3
Note C: Recovery of kappa from cell densities 3
Note D: Kappa from s(t) 4
Note E: Linear projection of velocities onto PCA embedding 4
Note F: Nonlinear Nyström projection for Diffusion maps 4
Note G: Processing of datasets 4
Note H: Simulation 6
Note I: processing of real datasets 7
Note J: Evaluation of inferred velocities in simulation data 8

References **9**

Note A: Scaling of unspliced counts

The RNA velocity protocol is based on separating spliced from unspliced molecules. There exists multiple counting software to quantify these molecules. Here, we will focus on the quantification rules as described in [1]:

- Molecules are counted as "spliced" if all of the reads in the set supporting a given molecule map only to exonic regions.
- Molecule are counted as "unspliced" if all, or at least one read in the set spans an exon-intron boundary, or is mapped to an intron for every compatible transcript models.
- Molecules are counted as "ambiguous" if some of the compatible transcript models have exonic mappings, and others intronic mappings. These counts are disregarded in further analyses.

Since unspliced RNA also contain exonic regions and we do not measure the whole length RNA we expect that some of the unspliced RNA will be misassigned to spliced. Additionally, if the sampling of molecules is biased for specific regions of the transcripts, s.a. for the 3' end, this will also affect the measured counts. In both these cases, we expect the measured ratio of unspliced to spliced to be affected. To illustrate the issues this can cause in the recovery of reaction rate parameters, we will first explain how the reaction rate parameters affect two aspects of the unspliced, spliced ratio: (1) the slope of the steady-state ratio and (2) the curvature of the phase trajectory in transient state.

Consider one gene with true parameters of reaction rate $\theta = (\kappa\alpha, \kappa\beta, \kappa\gamma)$. At up-regulation, the spliced and unspliced abundances can potentially reach their steady state in the limit

$$\begin{aligned}(u_\infty, s_\infty) &= (\frac{\alpha\kappa}{\beta\kappa}, \frac{\alpha\kappa}{\gamma\kappa}) \text{ , setting } \beta = 1 \\ &= (\alpha, \frac{\alpha}{\gamma})\end{aligned}$$

The slope of the steady state ratio is then given by γ, or $\frac{\kappa\gamma}{\kappa\beta}$. To understand which parameter affects the curvature of the phase trajectory in transient state, we rearrange the equation of $s(u)$. For simplicity, we focus on up-regulation:

$$
\begin{aligned}
s(u) &= (s_0 - \frac{\alpha}{\gamma} + \frac{\alpha - u_0}{\gamma - 1})(\frac{u-\alpha}{u_0 - \alpha})^{\gamma} + \frac{u-\alpha}{\gamma-1} + \frac{\alpha}{\gamma} \text{ , at up-regulation } (u_0, s_0) = (0,0) \\
&= (-\frac{\alpha}{\gamma} + \frac{\alpha}{\gamma-1})(\frac{u-\alpha}{-\alpha})^{\gamma} + \frac{u-\alpha}{\gamma-1} + \frac{\alpha}{\gamma} \\
&= (\frac{\alpha}{\gamma(\gamma-1)})(\frac{u-\alpha}{-\alpha})^{\gamma} + \frac{u-\alpha}{\gamma-1} + \frac{\alpha}{\gamma} \\
&= (\frac{1}{\gamma-1})(\frac{\alpha}{\gamma}(\frac{u-\alpha}{-\alpha})^{\gamma} + (u-\alpha)) + \frac{\alpha}{\gamma} \\
&= (\frac{1}{\gamma-1})(\frac{(-(u-\alpha))^{\gamma}}{\gamma\alpha^{\gamma-1}}) + (u-\alpha)) + \frac{\alpha}{\gamma} \text{ , setting } \frac{1}{\gamma-1} = c_1 \text{ and } \frac{\alpha}{\gamma} = c_2 \\
&= c_1(\frac{(-(u-\alpha))^{\gamma}}{\gamma\alpha^{\gamma-1}}) + (u-\alpha)) + c_2
\end{aligned}
$$

The relative contributions of $(u-\alpha)$ (linear) and $(-(u-\alpha))^{\gamma}$ (exponential) are weighted by $\gamma\alpha^{\gamma-1}$. In other words, the higher γ, the more $s(u)$ will be linear. This means that γ, or $\frac{\kappa\gamma}{\kappa\beta}$ determines both the steady-state slope and the curvature of the phase trajectory at transient state.

If the recovered values for unspliced, spliced counts are not proportional to the true values for each transcript, we cannot recover a γ that explains both the steady-state ratio and the curvature.

To account for this, we assume that the measurement follow $(\vec{u}_{obs}, \vec{s}_{obs}) \sim (\frac{\vec{u}}{m}, \vec{s})$, with m a constant scaling factor for that gene and $(\vec{u}, \vec{s})$ the unknown true unspliced and spliced counts for one cell.

Scaling of unspliced counts in scVelo

To account for the fact that the observed unspliced and spliced counts cannot be well described by the phase trajectory without scaling, scVelo [2] scales the measured unspliced counts s.t. they have the same variance as the spliced ones: $u_{scaled} = u_{obs} * \frac{\sigma(s_{obs})}{\sigma(u_{obs})}$. Scaling by $\frac{\sigma(s_{obs})}{\sigma(u_{obs})}$ in this way puts u_{scaled} on the same range of values as s_{obs}, which is only true for $\frac{\kappa\gamma}{\kappa\beta} \approx 1$.

Unbiased measurement of spliced and unspliced molecules

Learning m in addition to (α, β, γ) from unspliced, spliced measurements alone adds an additional layer of complexity. Ideally, we would count the molecules in an unbiased way, and thus not need to fit m. An alternative set of quantification rules would be given by:

- Molecules are counted as "spliced" if all of the reads in the set supporting a given molecule map only to exonic regions and at least one read overlaps an exon-exon boundary.
- Molecule are counted as "unspliced" if all, or at least one read in the set spans an exon-intron boundary, and no read overlaps an exon-exon boundary.
- Molecules are counted as "ambiguous" in three cases: (1) if all reads in the set map only to introns, (2) if all reads in the set map only to exons and none of the reads overlap an exon-exon boundary, or (3) if some of the compatible transcript models have exonic mappings, and others intronic mappings. These counts are disregarded in further analyses. Molecules that only align to either introns or exons need to be discarded as the length of the intronic region will influence the sampling of reads. Additionally, reads aligning only to exonic region could come both from spliced and unspliced molecules.

This set of rules only counts molecules with at least one read overlapping splicing sites, and thus will not inflate any of the counts. It is very likely that a lot of sequenced reads will not align to splice junctions, and will have to be discarded for RNA velocity analysis, but the resulting measurement would more accurately represent the true ratio of unspliced to spliced.

Note B: Expectation maximisation

Given $(\vec{u}_{obs}, \vec{s}_{obs}) \sim (\frac{\vec{u}}{m}, \vec{s})$ the vectors of length n of measured unspliced and spliced counts, we try to find a trajectory $s(u)$ specified by $\theta = (\alpha, \gamma, u_k)$ that best describes the observations. In practice, due to bias in the assignment of reads, the measured counts are often scaled by a factor m that needs to be inferred as well (see Note A), e.g. $\theta = (\alpha, \gamma, u_k, m)$ and we try to find the trajectory $s(u * m)$.

To infer (α, γ, u_k, m), we try to minimise the sum of cell-wise residuals (e_i) of the observed states to the fitted trajectory. Given $(p_i, s(p_i))$, the closest point to mu_i, s_i on the fitted trajectory, we have:

$$\begin{aligned}\text{cost}(\alpha, \gamma, u_k, m|\vec{u}_{obs}, \vec{s}_{obs}) &= \sum_i e_i \\ &= \sum_i ((mu_i - p_i)w)^2 + (s_i - s(p_i))^2\end{aligned}$$

, with $w = \sigma(\vec{s})/\sigma(\vec{u}m)$ a weighting factor such that distances in u and s are weighted equally.

To calculate the residuals, we need to find the points $(p_i, s(p_i))$. k_i is set to 1 if $\frac{nu_i}{s_i} > \gamma$ and 0 otherwise. The inference of p_i from $(u_i, s_i), (\alpha, \gamma, u_k, n)$ has no closed form solution, and calculation of all p_i by gradient descent while trying to find (α, γ, u_k, m) would be very computationally heavy. Because of this, during inference of (α, γ, u_k, m), we approximate p_i by computing $m = 100$ points $(p_j, s(p_j))$ with p_j in the range $(0, u_k)$ and assign p_i to the $(p_j, s(p_j))$ closest to the measured counts. In the last step of parameter inference, to get the final likelihood of the fitted parameters we calculate the true p_i from gradient descent.

The final likelihood of the fit for the gene is given by:

$$L(\alpha, \gamma, u_k, m|\vec{u}_{obs}, \vec{s}_{obs}) = \frac{1}{\sqrt{2\pi\sigma^2}} \exp(-\sum_i^n \frac{((mu_i - p_i)w)^2 + (s_i - s(p_i))^2}{2n\sigma^2})$$

Note C: Recovery of kappa from cell densities

For one gene after the parameter fitting procedure by EM, we recover the parameters of reaction rate $\theta = (\alpha, \beta, \gamma)$, the upscaling factor m as well as $t(i)$ the time assignment for cell i, and $u_{t(i)}$ the assigned unspliced counts for i (we use u here after upscaling the measured u i.e., corresponding to $m * u$ in the main text).

Therefore we can rewrite Equation 10 from the main text as:

$$\kappa n = -\frac{1}{\beta} \log \frac{u_{t(i)} - \alpha/\beta}{u_{t(j)} - \alpha/\beta} \tag{1}$$

$$\kappa d(i, j) = f(i, j) \tag{2}$$

Without measurement noise this equation should return the same value for any pairs of cells in the same transient state of transcription. To account for noise, we randomly sample pairs of cells i, j that are in the same transcriptional state, and compute $d(i, j)$ and $f(i, j)$ for each pair. Plotting d on the x-axis and f on the y-axis, the slope of the corresponding line gives us κ (see S2 Fig B). Towards the extremes if one (or both) of i, j is in steady-state and falsely assigned to a transient transcriptional state, f will be smaller than expected from equation 2, and we will have points right of the κ line on the d, f plot (see S2 Fig B). The κ line is still given by the left slope of the d, f plot. To recover κ, we fit a parallelogram to the points such that the area of the parallelogram is minimal while maximising the number of points in the parallelogram (see S2 Fig B), the κ-slope is then given by the left side of the parallelogram.

Note that the recovered κ for all genes are still off by a same constant factor c, that would relate the density to true time. Since this factor is constant for all κ, we can still relate velocities across genes.

Note D: Kappa from s(t)

Let Δt_{ij} be a measure of time that can be used to relate time between two states i, j across genes, with i before j in time. Consider one gene with true parameters of reaction rate $\theta = (\kappa\alpha, \kappa\beta, \kappa\gamma)$ and recovered parameters $\theta = (\alpha, \beta, \gamma)$.
The equation for spliced (s) counts as a function of time is $s(t) = s_0 \exp(-\kappa\gamma(t - t_0)) + \frac{\alpha}{\gamma}(1 - \exp(-\kappa\gamma(t - t_0))) + \frac{\alpha - \beta u_0}{\gamma - \beta}(\exp(-\kappa\gamma(t - t_0)) - \exp(-\kappa\beta(t - t_0)))$, where s_0, u_0 are the initial condition of the s and unspliced (u) counts at time t_0. This yields for two measurements from cell i and j:

$$s_j = s_i \exp(-\kappa\gamma\Delta t_{ij}) + \frac{\alpha}{\gamma}(1 - \exp(-\kappa\gamma\Delta t_{ij})) + \frac{\alpha - \beta u_i}{\gamma - \beta}(\exp(-\kappa\gamma\Delta t_{ij}) - \exp(-\kappa\beta\Delta t_{ij})) \quad (3)$$

Solving for $\kappa\Delta t_{ij}$ we get:

$$\kappa\Delta t_{ij} = -\frac{1}{\gamma} \log \frac{s_j - \alpha/\beta + \frac{\alpha - \beta u_j}{\gamma - \beta}}{s_i - \alpha/\beta + \frac{\alpha - \beta u_i}{\gamma - \beta}} \quad (4)$$

Note E: Linear projection of velocities onto PCA embedding

For projection of end of the velocity arrows (test set data points) onto the existing principal component analysis (PCA) of initial cell positions (training set), we use the weight matrix $W_{g\times k}$ that transformed the training points $S_{n\times G}$ into the PCA transformation $Y_{n\times k}$ of S on the first k principal components.
We have:

$$Y = SW \quad (5)$$

Since PCA is a linear transformation of gene space, we can apply the same transformation on the future cell states $S + \vec{V}$. The PCA-transformed future states are:

$$Y_{fut} = (S + \vec{V})W \quad (6)$$

The single-cell velocities can then be visualised on the first k principal components.

Note F: Nonlinear Nyström projection for Diffusion maps

In diffusion maps the embedding $Y_{n_{train}\times k}$ corresponds to the first k right eigenvectors $Y = [\psi_0, \ldots, \psi_k]$ of P. With the corresponding ordered eigenvalues Diagonal$(\Lambda) = \lambda_0 \geq ... \geq \lambda_k$, we have: $PY = Y\Lambda$. In this special case, the embedding matrix Y (eigenvectors) inversely scaled by the corresponding eigenvalues λ gives us the projection as:

$$Y_{test} = P'Y\Lambda^{-1} \quad (7)$$

Note G: Processing of datasets

Any velocity workflow starts with counting the spliced and unspliced reads (preprocessing). This is followed by several processing operations that either filter (remove genes or cells from the counts matrix) or manipulate the data (normalisation, imputation, etc.). Eventually the data is used for the downstream (velocity) analysis. Both preprocessing and processing have major effects on the outcome of the downstream analysis. Therefore it is essential to understand the implications of each step in the (pre)processing pipeline. After careful investigation of the scVelo processing pipeline [2], we have come to the conclusion that some processing steps should be updated to reduce the introduction of artefacts and to improve the recovery of the rate parameters α, β and γ. Below, we will describe our four-step processing pipeline, which is part of the κ-velo workflow, and give a detailed explanation of the implications of each step. Additionally, we will describe two filtering steps that follow the recovery of the rate parameters. S4 Fig gives a detailed overview of each step in the processing workflow and how the steps change the U and S counts matrices (middle) and the u-s phase portrait of individual genes (right).

Step 1: Select highly variable genes

To decrease the dimensions of the single-cell dataset we select only those genes that have a high variability. We use analytic Pearson residuals to identify the variability of each gene [3]. We prefer this method over other methods, because it recovers both low- and high-expression genes with high variance. The HVGs are identified using the spliced counts only. In S4 Fig we see that genes *Acly* and *Dpysl2* are selected, while gene *Gnaz* is filtered out.

Step 2: Select genes with high unspliced and spliced counts

After selecting the HVGs, we filter the genes once more to include only genes that have sufficient spliced and unspliced counts. In step 4 the spliced and unspliced counts for each gene in each cell are imputed using the nearest neighbours to decrease the noise in the measurements. Imputation can create artificial clouds of points in the unspliced/spliced portrait for some genes with extremely low unspliced and/or spliced counts, for which the dynamics cannot be reliably recovered (see S12 Fig). Therefore, we filter out all genes for which the spliced and/or unspliced counts are below a certain threshold. *Mt2* and *Ctsz* are examples of genes that have high variance, but extremely low unspliced counts (S4 Fig). The genes are therefore filtered out.

Step 3: Normalisation

In the processing pipeline of scVelo, unspliced and spliced counts are normalised separately. Normalisation is used to reduce the effect of count depth differences between cells [4]. Most commonly, the counts in each cell are divided by the total counts for that cell and multiplied by a scaling factor. Since spliced and unspliced counts derive from the same cells, the total counts of each cell can be calculated by summing both for each cell. Rather than normalising using separate total counts, the spliced and unspliced counts should be normalised by their combined total counts. This proves especially important in datasets where cell types have different ratios of total spliced to total unspliced counts, such as the erythroid lineage in the mouse gastrulation dataset published by Pijuan-Sala et al. [5] (S13 Fig A). In this erythroid branch Barile et al. found several genes with multiple rate kinetics (MURK genes) [6]. Those genes distinguish themselves in the unspliced/spliced phase portrait with a rapid increase of unspliced counts in the more differentiated cell types. Performing a combined normalisation in the original protocol, the curvature of these genes changes in the u-s phase portrait (S13 Fig B), indicating that this could be an artefact of normalisation. In S4 Fig it is shown how the normalisation changes the u and s counts of *Gcg* in the phase portrait.

Step 4: Imputation

The imputation step in the pipeline averages the spliced and unspliced counts of each cell using its nearest neighbours. Since we filtered out a large proportion of genes in step 1 and step 2, the PCA used for calculating the nearest neighbours is recalculated. For the computation of the PCA coordinates, only the spliced counts are used. For PCA calculation, the spliced counts are scaled to stabilise the variances of the genes. S4 Fig demonstrates how imputation changes the u and s counts of *Gcg*, *Nnat* and *Ptov1*.

Step 5: Select genes with high likelihood

During the recovery of the parameters, each gene is assigned a likelihood, which indicates how well a gene is described by the recovered phase trajectory. The genes can then be ranked according to likelihood of the fit. Only genes with a likelihood above a certain threshold are used for the downstream velocity analysis and embedding. Similar to step 2, the used threshold depends on the dataset. The more genes are included, the more details the velocity embedding might reveal. On the other hand, if we include too many genes with poorly recovered dynamics, the more technical noise is introduced in the datasets. S4 Fig shows the recovered dynamics of *Gcg*, *Nnat* and *Dnmt3a*. The phase trajectory of *Gcg* and *Nnat* matches the observed u and s counts very well. The counts of *Dnmt3a* however do

not seem to be very well captured by the phase trajectory. With a likelihood of 0.3504 this gene is removed.

Step 6: Select genes matching known cluster order

To recover the parameters, κ-velo fits an upregulation, a downregulation, or both, to the u and s counts of each gene (upregulation: blue curve in S4 Fig, downregulation: orange curve in S4 Fig). However, in some cases the order of cell types in the recovered up-/downregulation does not match the known biological order of those cell types. For example, genes *Gcg* and *Dock11* both show a phase portrait where the blue cell type (alpha cells) has on average the highest u and s counts. Given that alpha cells are preceded by several other precursor cell types during differentiation (in this dataset), the high u and s counts in this cell type can only be explained by an upregulation. That is to say, a downregulation can never start at the most differentiated cluster. In the case of *Dock11* that means that the gene has to be removed, because a downregulation is falsely assigned to these cells. In order to filter those genes we provide the user of κ-velo with the option of entering the order of clusters in the dataset if (some part of) the order is known. More precisely, the user can list pairs of directly related cell types where it is known which of the two is the preceding ("parent") cluster and which one is the succeeding ("child") cluster. For both clusters we calculate the average unspliced and spliced count. We position both averages on the phase portrait. Based on the position of the child cluster with regards to the parent cluster, we decide whether the cells between the two averages (of both respective clusters) are part of an upregulation or downregulation. By repeating this for all parent-child cluster pairs we assign cells to upregulation, downregulation or ambiguous when we can not determine the state with certainty. Afterwards, we compares these state assignments with the state assignments from the recovery of the dynamics. The user defines a percentage of cells that have to match the original states in order for the gene to be included in the downstream analysis. In Figure S4 Fig we see how this filtering steps removes genes *Dock11* and *Ghrf*, because the clustering ordering could only be the assigned to an upregulation.

Comment 1: Log transformation

Long-read sequencing methods such as smart-seq yield a better disentanglement of u and s counts, hence seem more suitable for RNA-velocity analysis. Whereas several current single-cell methods (PCA, t-SNE, nearest neighbours search, etc) are usually used for non-UMI data only after log-transformation, the u-s dynamical equations used for parameter estimation in the RNA-velocity framework are defined for raw counts (e.g. by using $u_0 = 0$ and $s_0 = 0$ as the initial point in the u-s phase portraits). Most likely, this is also the reason that scVelo does not log transform the spliced and unspliced counts that are used for recovery of the dynamics. Both of scVelo's functions `scvelo.pp.filter_and_normalize` and `scvelo.pp.log1p` do not log-transform the u, s layers used for parameter recovery. To avoid any inconsistency, our processing pipeline does not include any log transformation.

Comment 2: Batch correction

With the technological advance of single-cell technologies, more and more single-cell RNA seq datasets may be compiled from multiple experiments. In most multi-sample cases, a batch correction is essential to recover the biological variation, while removing batch-effects [7]. However, batches may affect unspliced and spliced counts differently. Therefore it is important the be cautious when using any batch correction, and to always check that the recovered dynamics do not contain batch- or batch correction-related artefacts.

Note H: Simulation

For the simulation we randomly sampled G log-normally distributed parameters $\theta = (\alpha, \beta, \gamma)$ with $\log(\theta) \sim N(\mu, \Sigma)$, $\mu = (1, 0.2, 0.05)$ and $\Sigma_{ii} = 0.04, \Sigma_{ij,i\neq j} = 0.04 * 0.02$. The parameters are scaled by a scaling factor κ: $\theta = (\kappa\alpha, \kappa\beta, \kappa\gamma)$. The n cells follow a uniformly distributed hidden true time

t, with cell-specific time points $(t_1, ..., t_n)$. The unspliced and spliced counts for genes are simulated following

$$u(t) = u_0 \exp(-\beta\tau) + \frac{\alpha}{\beta}(1 - \exp(-\beta\tau))$$
$$s(t) = s_0 \exp(-\gamma\tau) + \frac{\alpha}{\gamma}(1 - \exp(-\gamma\tau)) + \frac{\alpha - \beta u_0}{\gamma - \beta}(\exp(-\gamma\tau) - \exp(-\beta\tau))$$

with $\tau = t - t_0 - t_s$, t_0 the time of transcriptional switch (on to off) and t_s the time of transcriptional start (off to on). The time point of transcriptional start for a gene was chosen inversely to the speed of the gene so that all genes reach the end of transcription at the same time. This results in a trajectory with high plasticity at the beginning when fast genes are not yet activated and lower plasticity at the end when all genes are in transient state.
Random normal noise was added to the unspliced and spliced counts.

Note I: processing of real datasets

Here, we give a more detailed description of all parameter and threshold settings for each of the datasets. As κ-velo and eco-velo are based on different assumptions, we describe the processing pipeline for both separately. The description here matches the code that is available on our Github page https://github.com/bjbouman/cell-state-velocities.

Processing pancreas endocrinogenesis dataset

Processing κ-velo: - Selecting HVGs: top 5000 HVGs selected - Selecting genes with high s and u counts: 406 genes selected (threshold > 4 counts) - Normalisation: L1 normalisation using sum of u and s counts as size vector - Imputation: using 15 first principal components and 30 nearest neighbours - Selecting genes with high likelihood: 374 genes selected (likelihood > 0.4) - Selected genes matching known cluster order: 134 genes selected

Processing eco-velo: - Selecting HVGs: top 5000 HVGs selected - Selecting genes with high s and u counts: 664 genes selected (threshold > 3 counts) - Normalisation: L2 normalisation using separate size vectors - Log-transformation - Selection of MNNs: neighbourhood size k=50 - Top 5 MNNs used for velocity vector

Processing murine hematopoiesis (HSPC) dataset

Before processing the HSPC dataset for recovering the velocities, we first recovered five of the main populations in the HSPC compartment: the hematopoietic stem cells (HSCs), erythroid progenitors, eosinophil/basinophil progenitors, megakaryocyte progenitors and myeloid progenitors. We combined the spliced and unspliced counts for each cell. Afterwards, we L2 normalised and scaled the data. We used a curated set of gene markers to score all the cells (set of marker genes available on our Github page under datasets/HSPC). We compared our assignments with the cell type annotations in the original dataset (see S18 Fig). Additionally, to verify the stem and progenitor populations, we calculated a stemness score for each cell. We took a set of curated genes associated with the HSPC compartment published by [8]. After scaling the dataset, we scored all cells for the average expression of these genes, compared to an equal-sized set of randomly selected genes.

Processing κ-velo: - Selecting HVGs: top 5000 HVGs selected - Selecting genes with high s and u counts: 531 genes selected (threshold > 6 counts) - Normalisation: L1 normalisation using sum of u and s counts as size vector - Imputation: using 15 first principal components and 30 nearest neighbours - Selecting genes with high likelihood: 340 genes selected (likelihood > 0.5) - Selected genes matching known cluster order: 137 genes selected

Processing eco-velo: - Selecting HVGs: top 2000 HVGs selected - Selecting genes with high s and u counts: 266 genes selected (threshold > 6 counts) - Normalisation: L2 normalisation using separate size vectors - Log-transformation - Selection of MNNs: neighbourhood size k=50 - Top 5 MNNs used for velocity vector

Processing chromaffin dataset - κ-velo

Processing κ-velo: - Selecting HVGs: top 2000 HVGs selected - Selecting genes with high s and u counts: 1894 genes selected (threshold $>$ 15 counts) - Normalisation: L1 normalisation using sum of u and s counts as size vector - Imputation: using 15 first principal components and 15 nearest neighbours - Selecting genes with high likelihood: 606 genes selected (likelihood $>$ 0.7) - Selected genes matching known cluster order: 112 genes selected

Processing eco-velo: - Selecting HVGs: top 500 HVGs selected - Selecting genes with high s and u counts: 474 genes selected (threshold $>$ 15 counts) - Normalisation: L2 normalisation using separate size vectors - Log-transformation - Selection of MNNs: neighbourhood size k=30 - Top 5 MNNs used for velocity vector

Processing erythroid dataset

The erythroid lineage of the murine gastrulation was processed using two different pipelines. First, we used the exact processing pipeline reported in [6]. Then we used our κ-velo processing pipeline using the following settings:

Processing κ-velo: - Selecting HVGs: top 5000 HVGs selected - Selecting genes with high s and u counts: 654 genes selected (threshold $>$ 3 counts) - Normalisation: L1 normalisation using sum of u and s counts as size vector - Imputation: using 15 first principal components and 30 nearest neighbours

Note J: Evaluation of inferred velocities in simulation data

We use three measures to evaluate the difference between two vectors $\vec{v}_t$ and $\vec{v}$ in PCA space. The cosine similarity $\frac{\vec{v}_t \times \vec{v}}{\|\vec{v}_t\| \|\vec{v}\|}$ is the cosine of the angle between the two vectors. The difference in vector length is given by as $\|\vec{v}_t\| - \|\vec{v}\|$. The norm of the errors is given by $\|\vec{v}_t - \vec{v}\|$.

References

1. La Manno G, Soldatov R, Zeisel A, Braun E, Hochgerner H, Petukhov V, et al. RNA velocity of single cells. Nature. 2018;560(7719):494–498. doi:10.1038/s41586-018-0414-6.

2. Bergen V, Lange M, Peidli S, Wolf FA, Theis FJ. Generalizing RNA velocity to transient cell states through dynamical modeling. Nature Biotechnology. 2020;38(12):1408–1414. doi:10.1038/s41587-020-0591-3.

3. Lause J, Berens P, Kobak D. Analytic Pearson residuals for normalization of single-cell RNA-seq UMI data. Genome biology. 2021;22(1):1–20.

4. Luecken MD, Theis FJ. Current best practices in single-cell RNA-seq analysis: a tutorial. Mol Syst Biol. 2019;15(6):e8746.

5. Pijuan-Sala B, Griffiths JA, Guibentif C, Hiscock TW, Jawaid W, Calero-Nieto FJ, et al. A single-cell molecular map of mouse gastrulation and early organogenesis. Nature. 2019;566(7745):490–495.

6. Barile M, Imaz-Rosshandler I, Inzani I, Ghazanfar S, Nichols J, Marioni JC, et al. Coordinated changes in gene expression kinetics underlie both mouse and human erythroid maturation. Genome Biol. 2021;22(1):197.

7. Tran HTN, Ang KS, Chevrier M, Zhang X, Lee NYS, Goh M, et al. A benchmark of batch-effect correction methods for single-cell RNA sequencing data. Genome Biol. 2020;21(1):12.

8. Giladi A, Paul F, Herzog Y, Lubling Y, Weiner A, Yofe I, et al. Single-cell characterization of haematopoietic progenitors and their trajectories in homeostasis and perturbed haematopoiesis. Nature cell biology. 2018;20(7):836–846.
