# Supplementary figures and images for: Towards reliable quantification of cell state velocities
Source: PLoS Comput Biol. 2022 Sep 28;18(9):e1010031. doi: 10.1371/journal.pcbi.1010031 (PMC9550177; doi:10.1371/journal.pcbi.1010031)

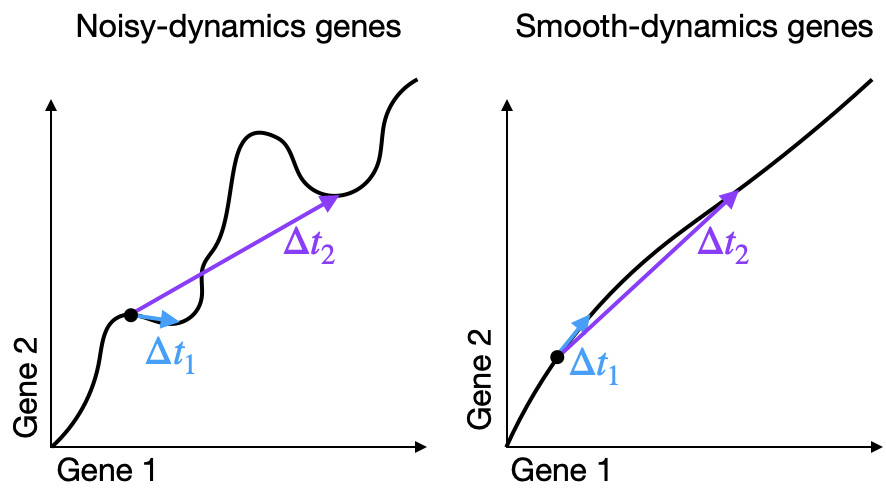

Supplement: S1 Fig — On the left is the example of two noisy genes: the average velocity over Δt1 is very different from the average velocity over Δt2. For smooth gene dynamics as shown on the right, the average velocities are more similar. (TIFF) [file pcbi.1010031.s003.tiff]

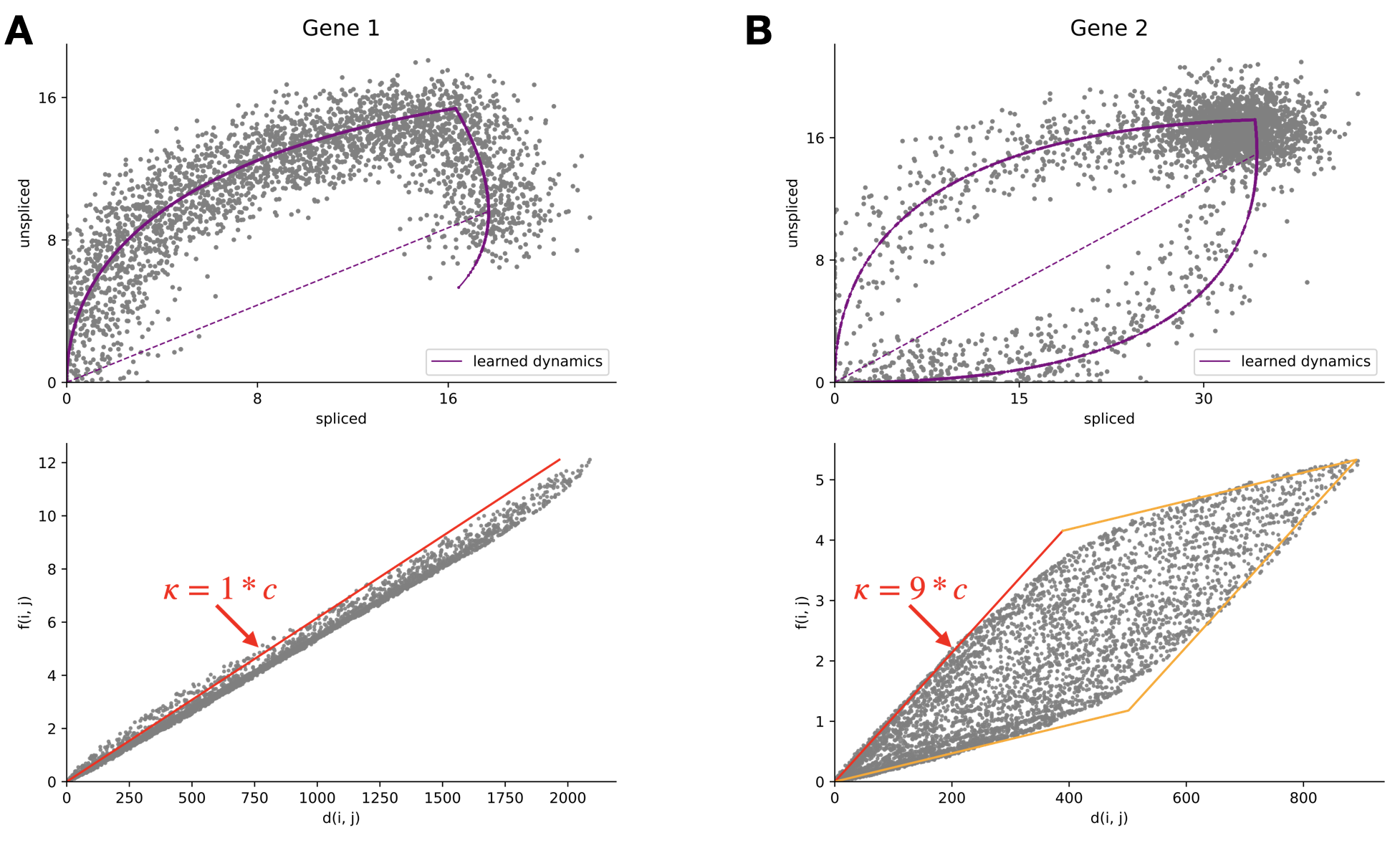

Supplement: S2 Fig — c = 10−3 is a constant scaling factor. The two simulated genes have the same reaction parameters θ but those for gene 2 are scaled by 10. (A) a slow gene, where no cells are in steady-state. The slope of the line gives us κg1 directly. (B) A fast gene, where a lot of cells are in steady-state. The slope of the red line gives us κg2. (TIFF) [file pcbi.1010031.s004.tiff]

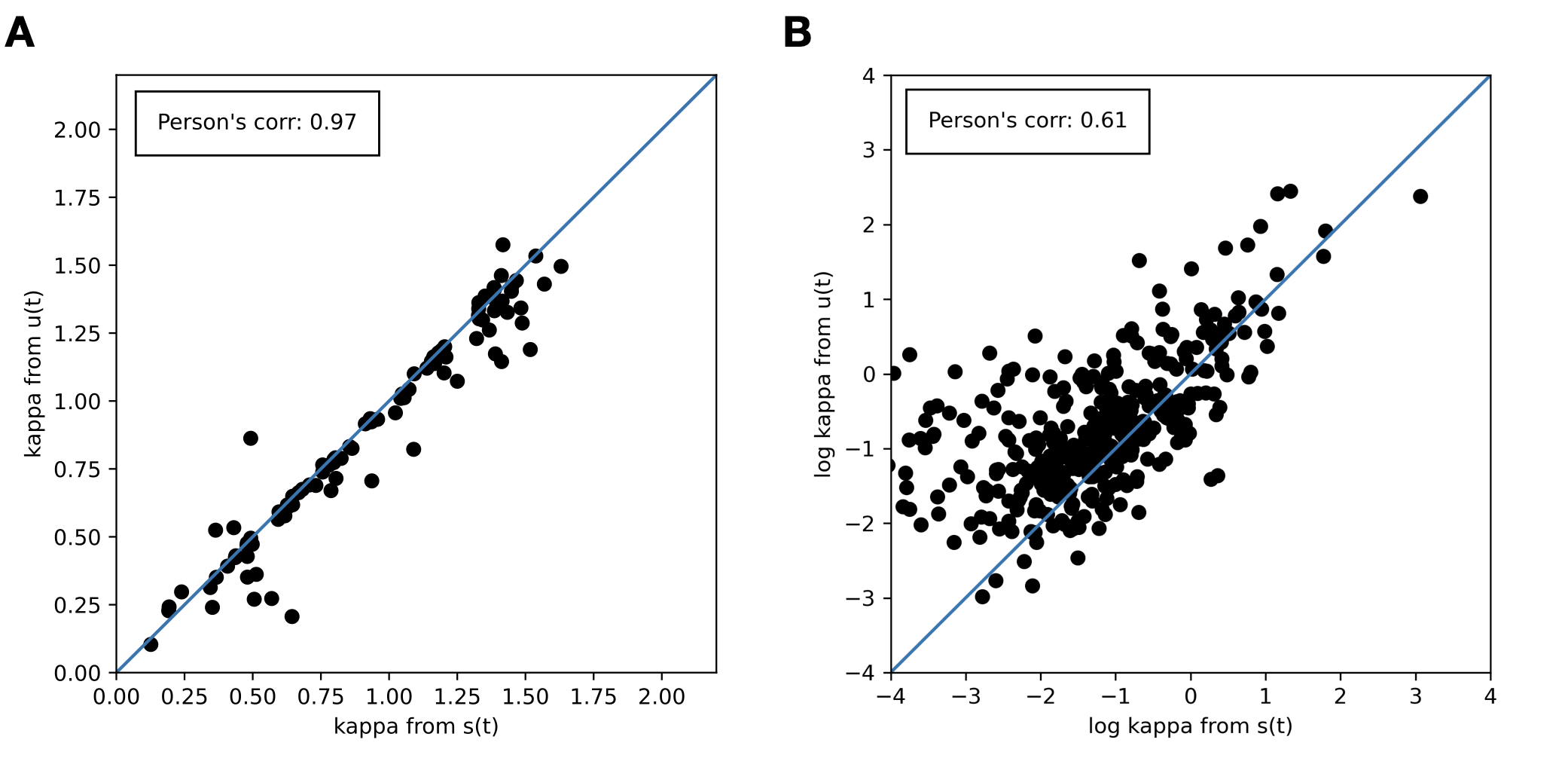

Supplement: S3 Fig — (A) On simulation; the simulation is the same as in main Fig 3. (B) On the pancreas endocrinogenesis dataset. (TIFF) [file pcbi.1010031.s005.tiff]

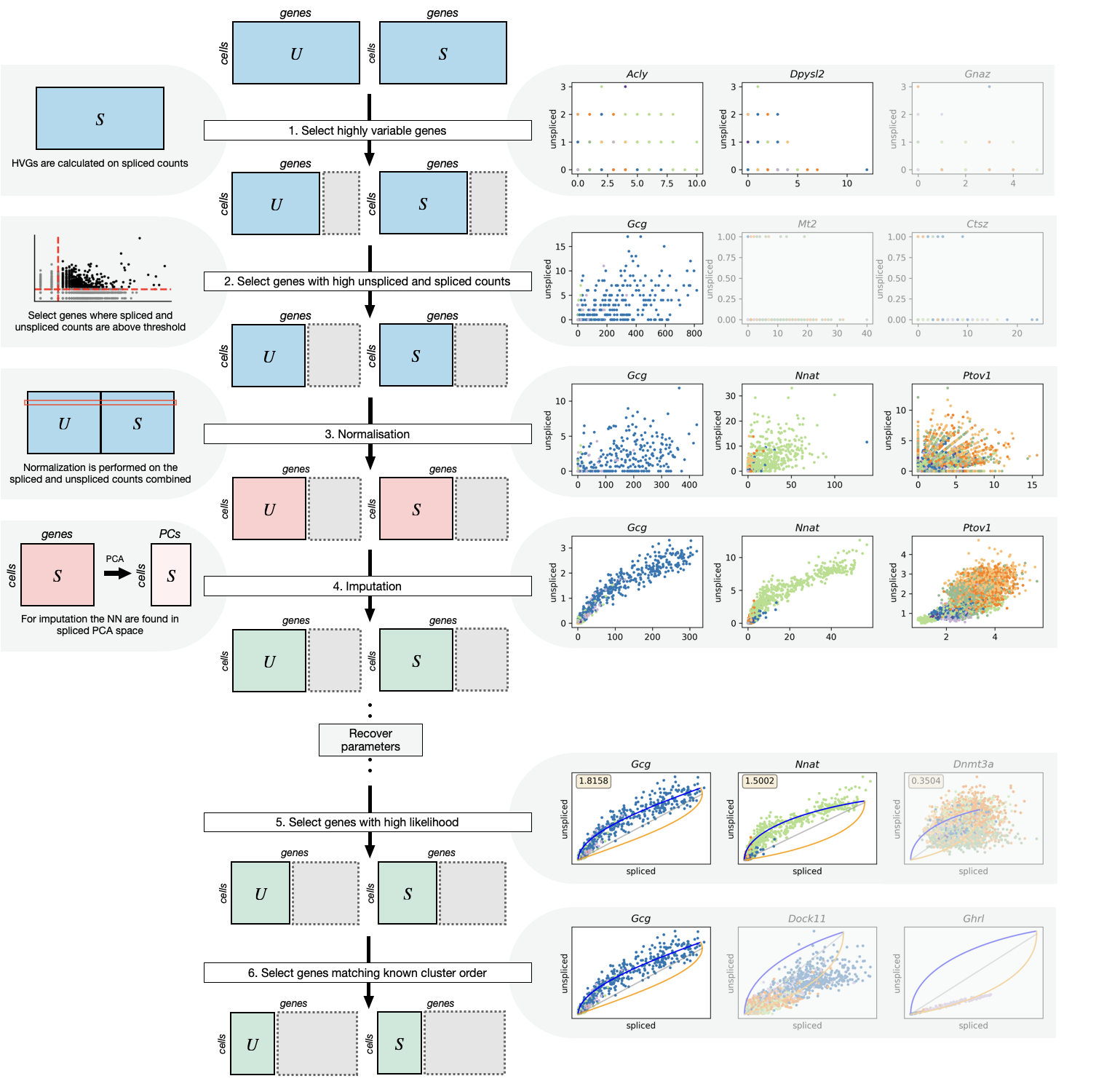

Supplement: S4 Fig — In the middle, a schematic representation of how the spliced and unspliced matrices change during each step is shown. A size reduction of the coloured area indicates a filtering step where the number of genes are reduced. A change in colour represent a data manipulation, which does not changes the number of cells or genes, but changes the values in the matrix. On the left, some extra information is provided for some of the processing steps. More detailed information can be read in Note G in S1 Appendix. On the right, the u-s phase portraits of several example genes are shown to demonstrate how the different steps change the phase portraits, as well as which kind of genes are selected or removed in the filtering steps. Each of the genes is selected from the pancreas endocrinogenesis dataset that is analysed in main Fig 4. (TIFF) [file pcbi.1010031.s006.tiff]

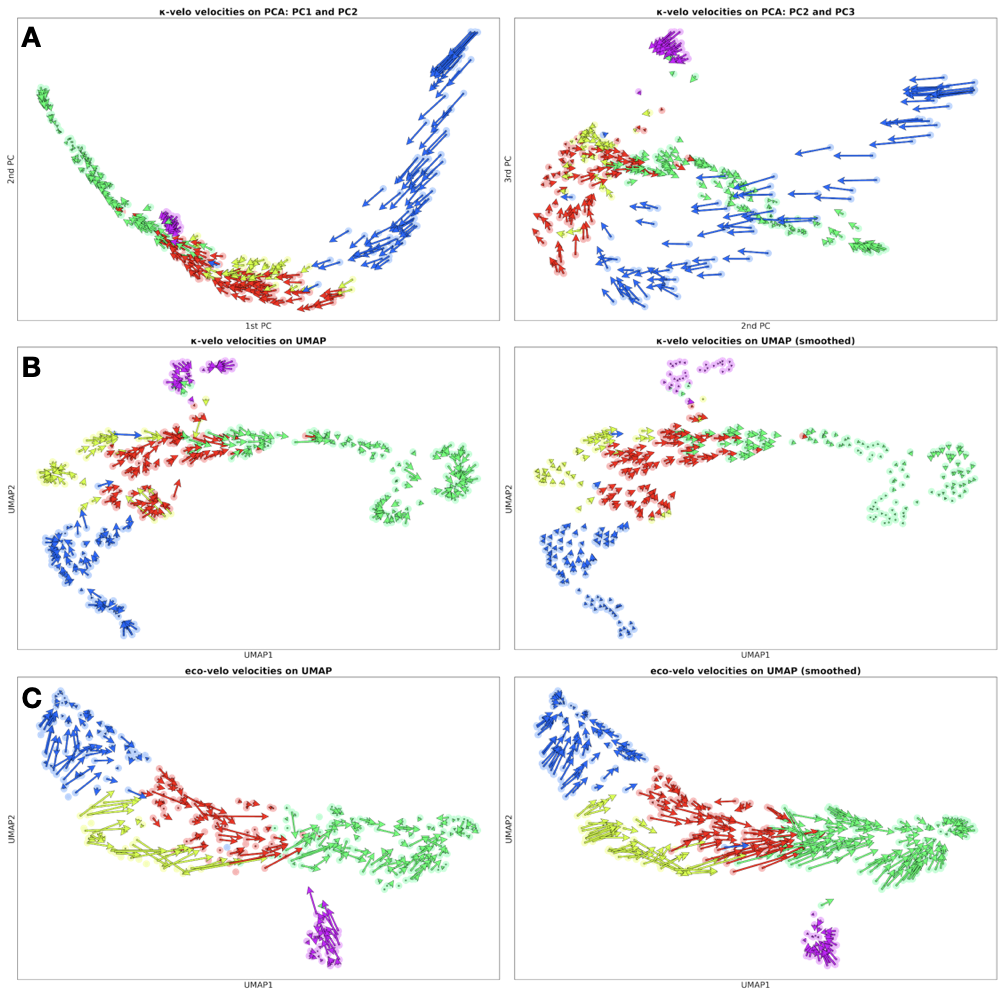

Supplement: S5 Fig — The chromaffin dataset includes Schwann cell precursors (SCPs) (blue) differentiating into chromaffin cells (green). In the original paper, the purple cluster was identified as symphatoblasts and the yellow and red cluster as “bridge” cells [22]. (A) κ-velo applied on chromaffin dataset using PCA embedding for visualisation. Principal component (PC) 1 and 2 left and PC 2 and 3 right. (B) κ-velo applied on chromaffin dataset using UMAP embedding for visualisation (left: raw vector visualisation, right: smoothed vector visualisation). (A) and (B) show that κ-velo correctly captures the differentiation from SCPs into chromaffin cells. Interestingly, there also seems to be a more committed differentiation in the bridge cells than the SCPs in the beginning of the manifold. (C) eco-velo applied on chromaffin dataset using UMAP embedding for visualisation (left: raw vector visualisation, right: smoothed vector visualisation). (TIFF) [file pcbi.1010031.s007.tiff]

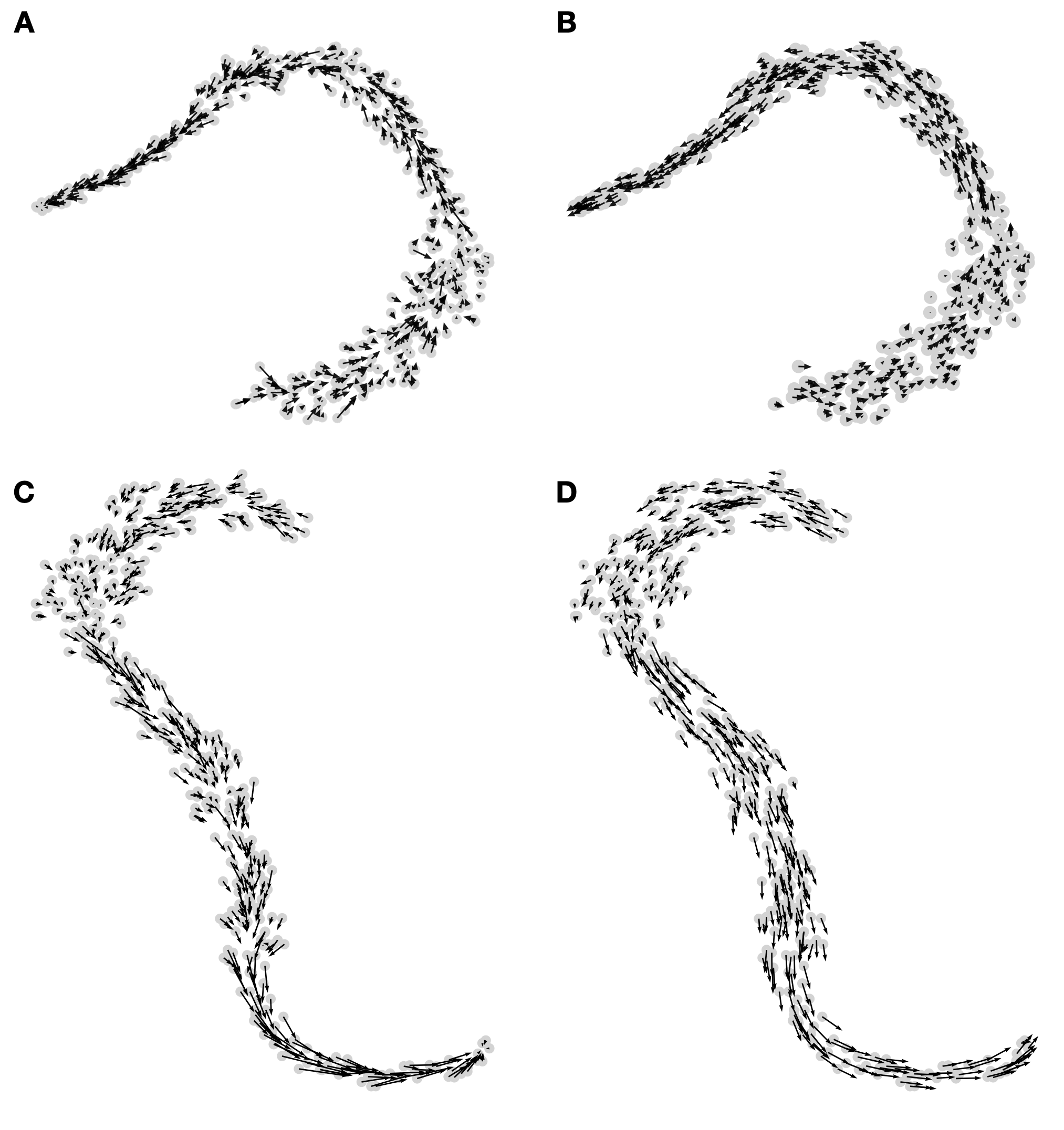

Supplement: S6 Fig — We compare our projection approach (left column) to scVelo’s [2] (right column) projection for t-SNE [16] in (A) and (B) and UMAP [17] in (C) and (D). (TIFF) [file pcbi.1010031.s008.tiff]

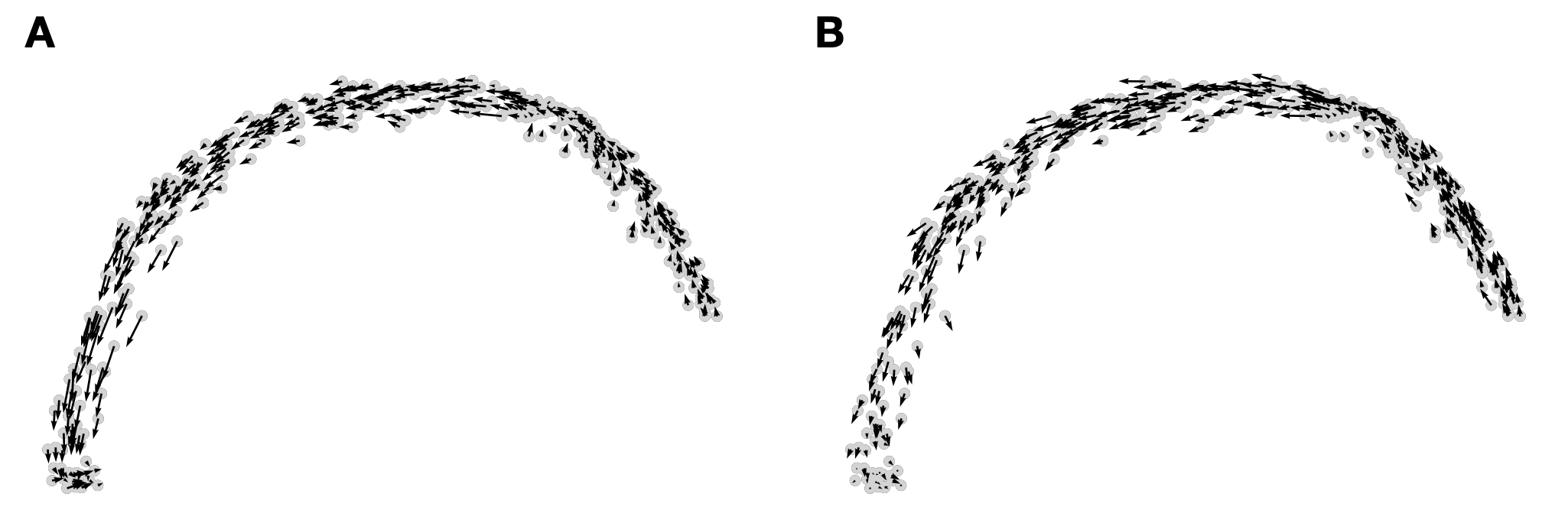

Supplement: S7 Fig — We compare our projection approach in A to scVelo’s [2]’s projection in B. (TIFF) [file pcbi.1010031.s009.tiff]

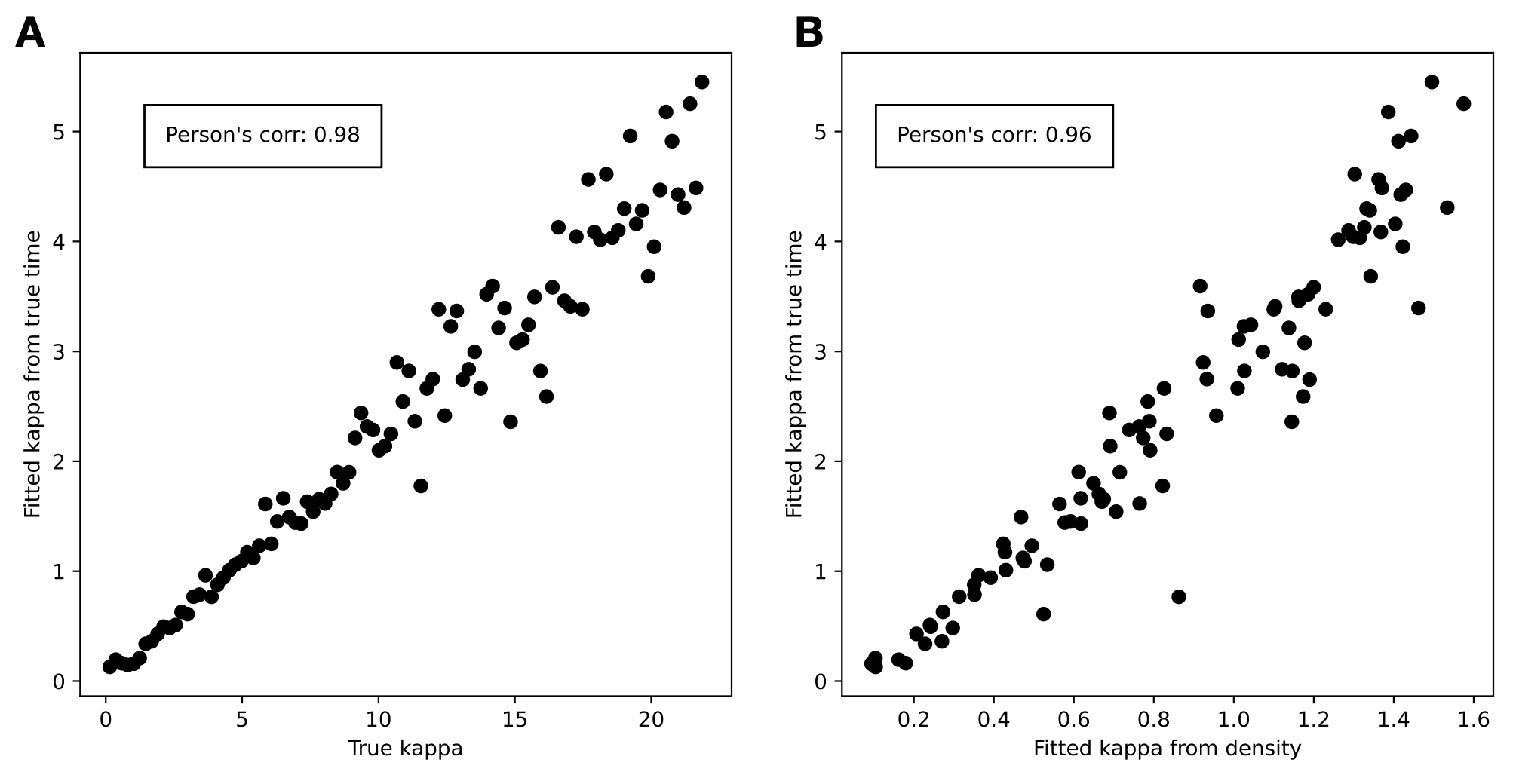

Supplement: S8 Fig — The simulation is the same as in main Fig 3. The factors are recovered similarly to the density approach described in Note C in S1 Appendix, except that d(i, j) is calculated from ti the true simulated time of cell i: d(i, j) = |(ti − tj)|. Plotting d on the x-axis and f on the y-axis, the slope of the corresponding line gives us κ. Here, since we have true time, we do not need to exclude steady-states. (A) Comparison of the scaling factors recovered from true time to the true simulated factors. Note that here the range of recovered scaling factors is equivalent to the true factors because they were recovered from true time and not from a proxy of time that might be off by some constant factor. (B) Comparison of the factors recovered from the density approach to the factors recovered from true time. (TIFF) [file pcbi.1010031.s010.tiff]

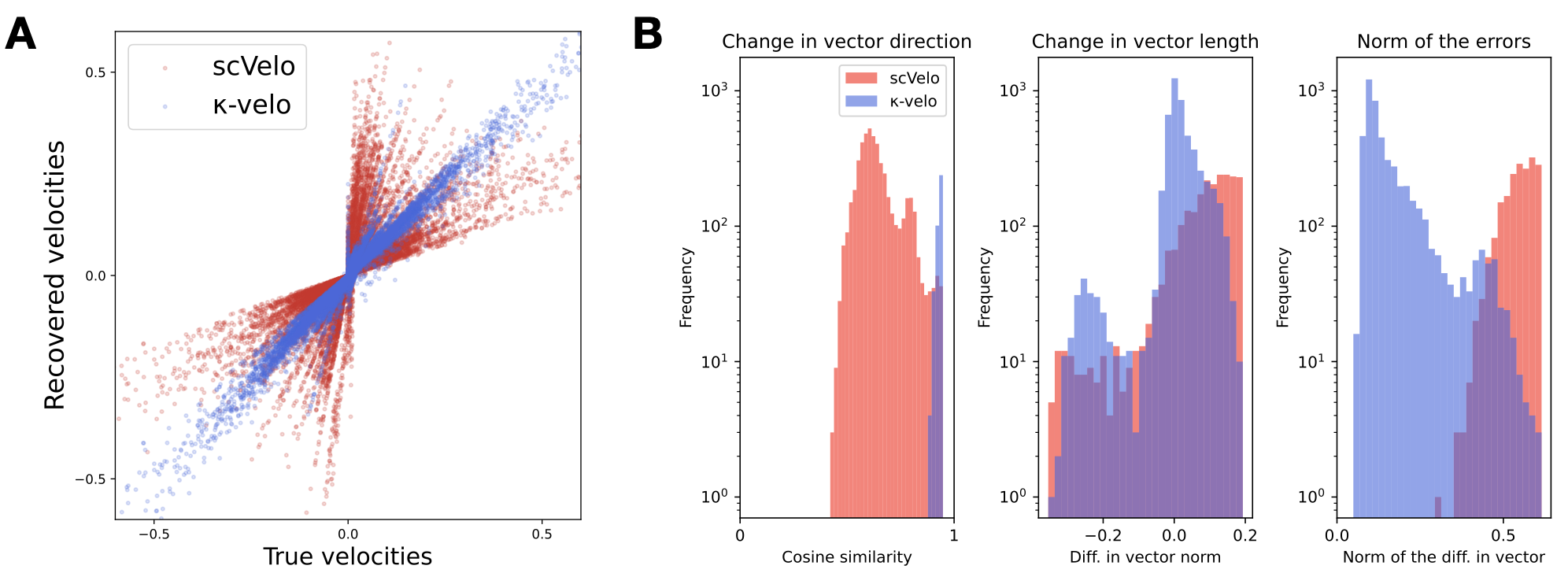

Supplement: S9 Fig — (A) High-dimensional velocity vector. One point represents a velocity for one cell for one gene. (B) We evaluate differences between true high-dimensional velocities and recovered velocities. We return the change in direction (cosine similarity), length (difference in vector norm) and the overall norm of the errors between real velocities and κ-velo velocities (in blue), or scVelo velocities (in red). To make the length comparable, the vectors high-dimensional vectors are normalised to have equal variance. Note the log-scale for frequency. (TIFF) [file pcbi.1010031.s011.tiff]

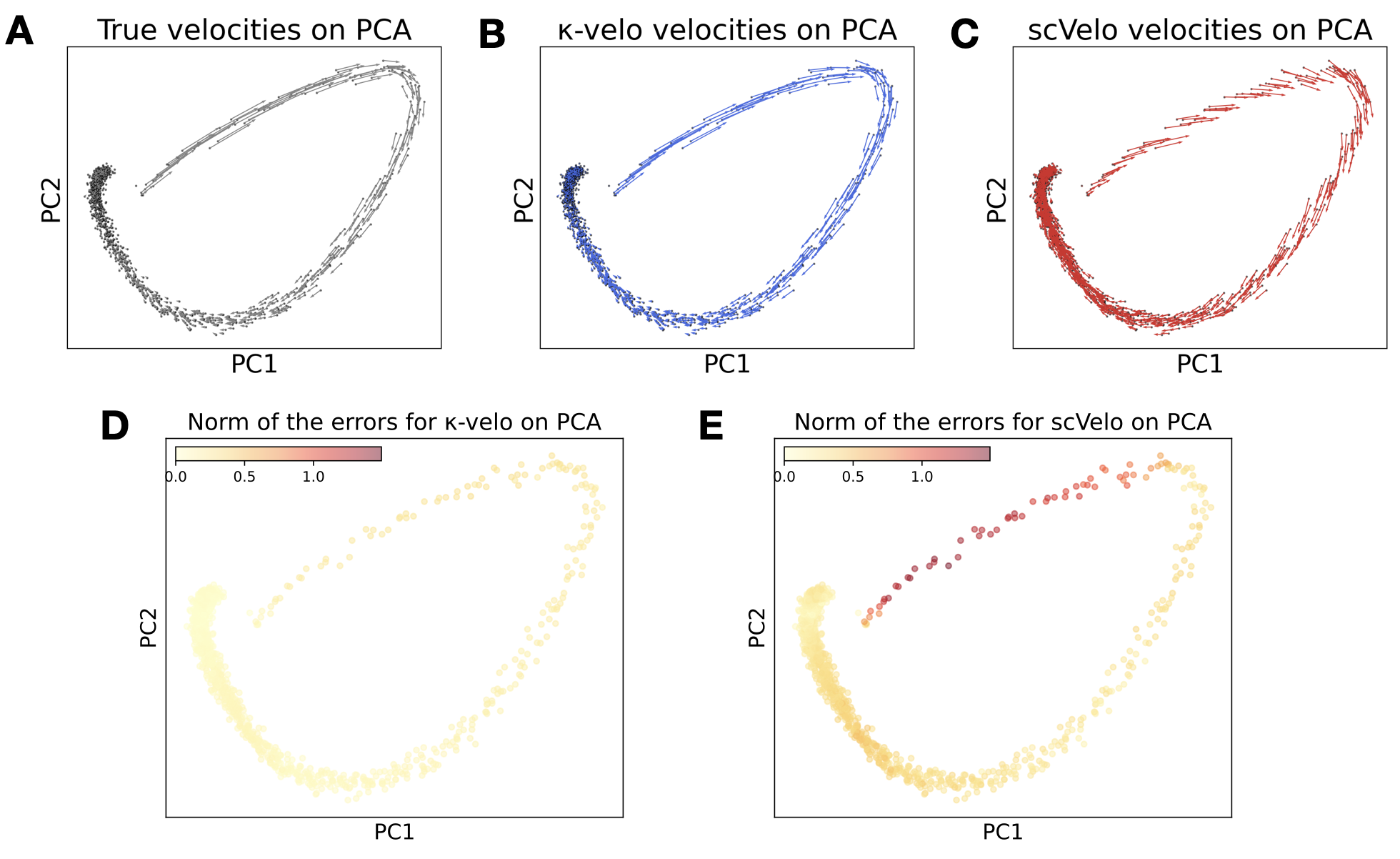

Supplement: S10 Fig — (A) Real simulated velocities (B) velocities recovered by κ-velo and (C) velocities recovered by scVelo projected on PCA. Cells on PCA coloured by norm of the errors between real velocities and (D) κ-velo velocities, or (E) scVelo velocities. (TIFF) [file pcbi.1010031.s012.tiff]

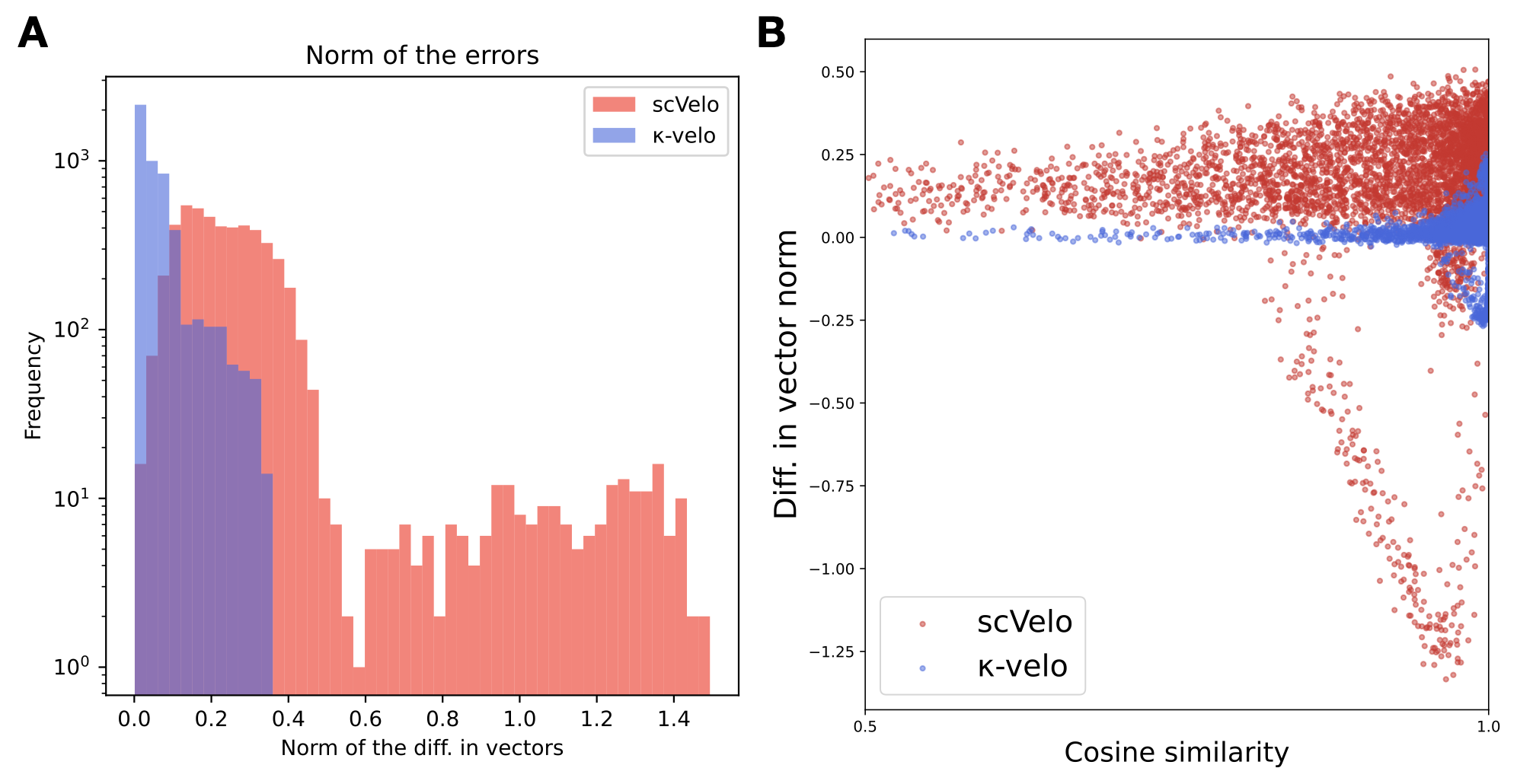

Supplement: S11 Fig — (A) Norm of the errors: ‖v→t-v→r‖ with v→t the true 2D velocity vector on PCA and v→r the recovered vector. (B) Change in direction (cosine similarity) and length (difference in vector norm: ‖v→t‖-‖v→r‖) for each cell in PCA space. (TIFF) [file pcbi.1010031.s013.tiff]

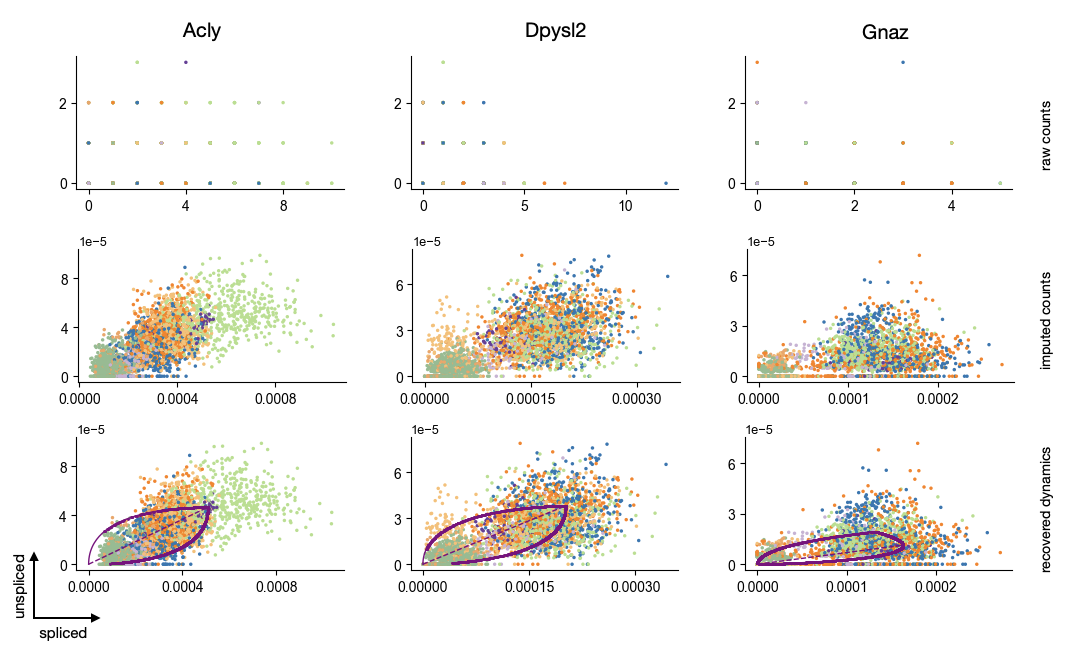

Supplement: S12 Fig — The u-s phase portrait of Acly, Dpysl2 and Gnaz (from the pancreas endocrinogenesis dataset), which are all genes with insufficient unspliced counts. Here, we show how scVelo would recover the dynamics if these genes were not filtered out. (TIFF) [file pcbi.1010031.s014.tiff]

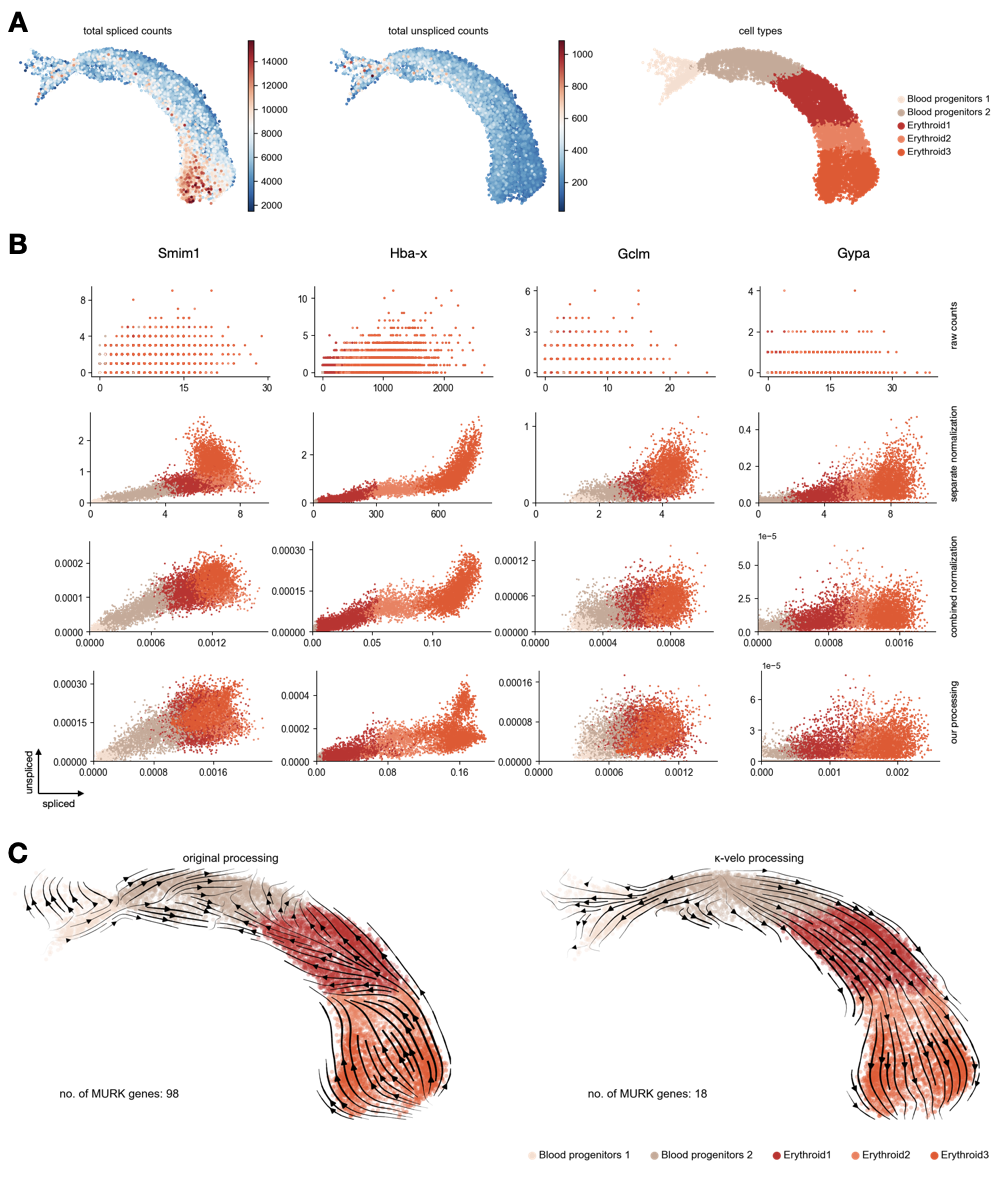

Supplement: S13 Fig — The scRNA-seq dataset on the erythroid lineage of mouse gastrulation [21] has been described in the context of RNA velocity by Barile et al. [6]. Here, we show that the subset has a varying ratio of total unspliced to total spliced counts in different cell types (A). This results in artefacts when using the standard scVelo processing pipeline (U and S normalised separately) (B, second row). Those artefacts are mostly resolved by normalising U and S combined (B, third row), which is part of the κ-velo processing workflow (B, last row). Using the κ-velo processing workflow fixes some of the reported de-differentiation (C). (TIFF) [file pcbi.1010031.s015.tiff]

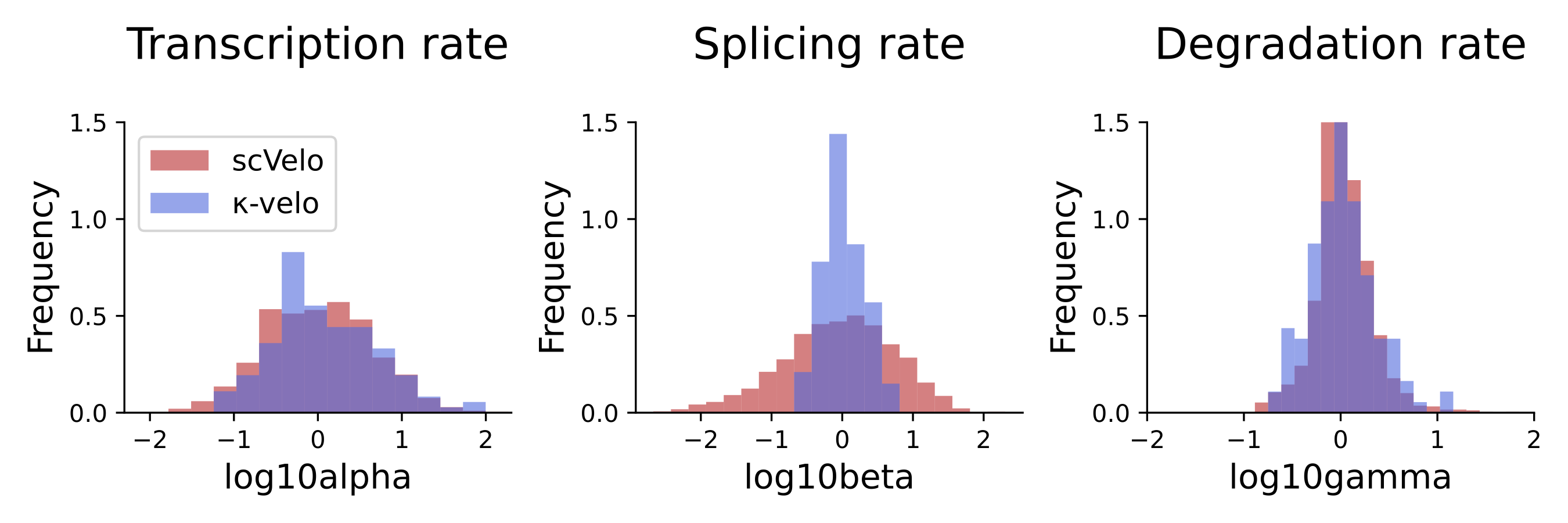

Supplement: S14 Fig — Range of transcription rate α, splicing rate β, and degradation rate γ estimated by scVelo (in red) and κ-velo (in blue). (TIFF) [file pcbi.1010031.s016.tiff]

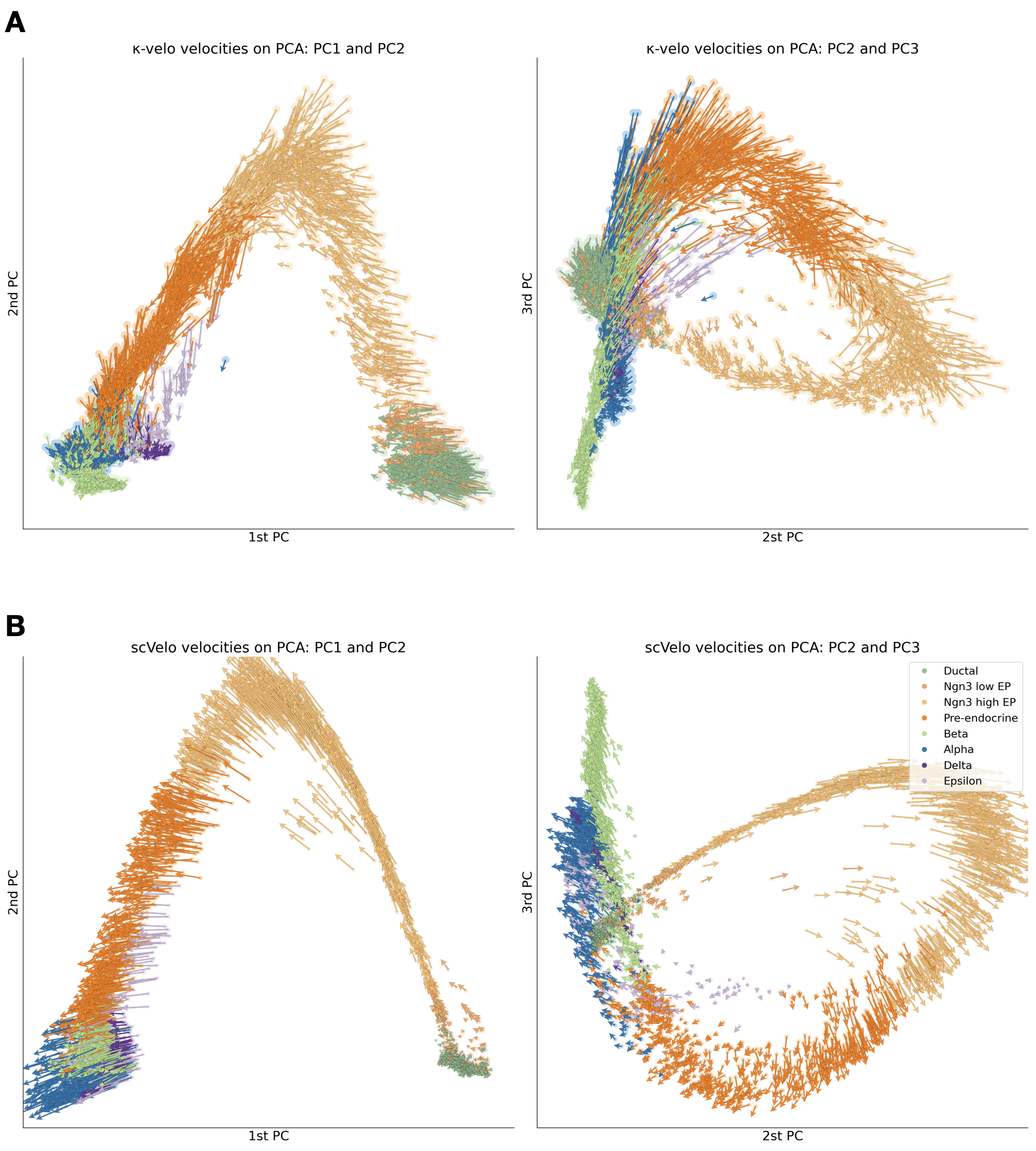

Supplement: S15 Fig — (A) Velocities returned by κ-velo projected on PCA embedding of spliced counts. (B) Velocities returned by scVelo projected on PCA embedding of spliced counts. We note that the gene space used is different for the two methods, as they have different criteria for gene selection. scVelo uses 1809 genes, while κ-velo uses 134. (TIFF) [file pcbi.1010031.s017.tiff]

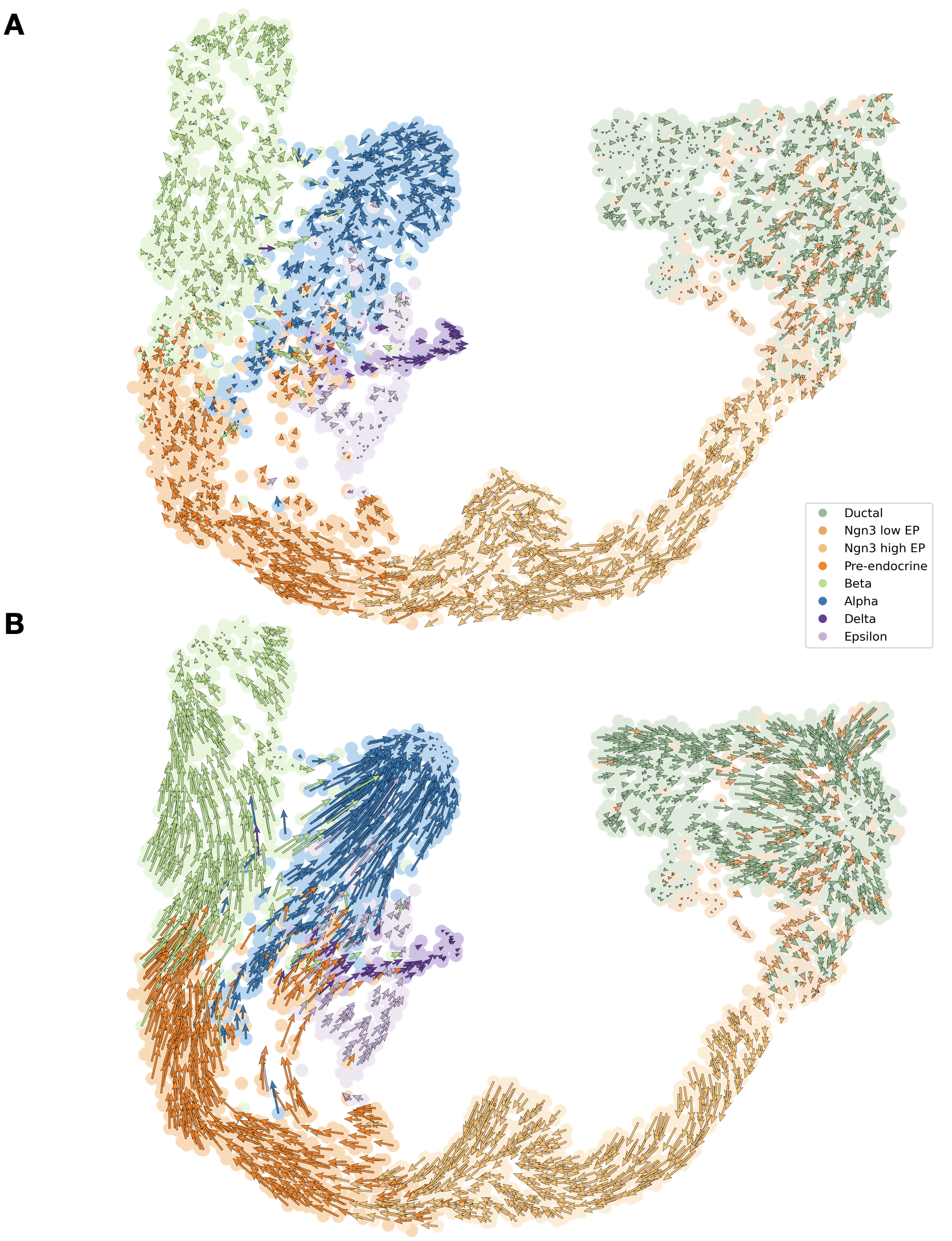

Supplement: S16 Fig — The two UMAPs compare (A) smoothed scVelo velocities projected by Nyström projection and (B) smoothed κ-velo velocities projected by Nyström projection. Velocities were smoothed by averaging over the 30 nearest neighbours. Neighbourhoods are calculated in S space. (TIFF) [file pcbi.1010031.s018.tiff]

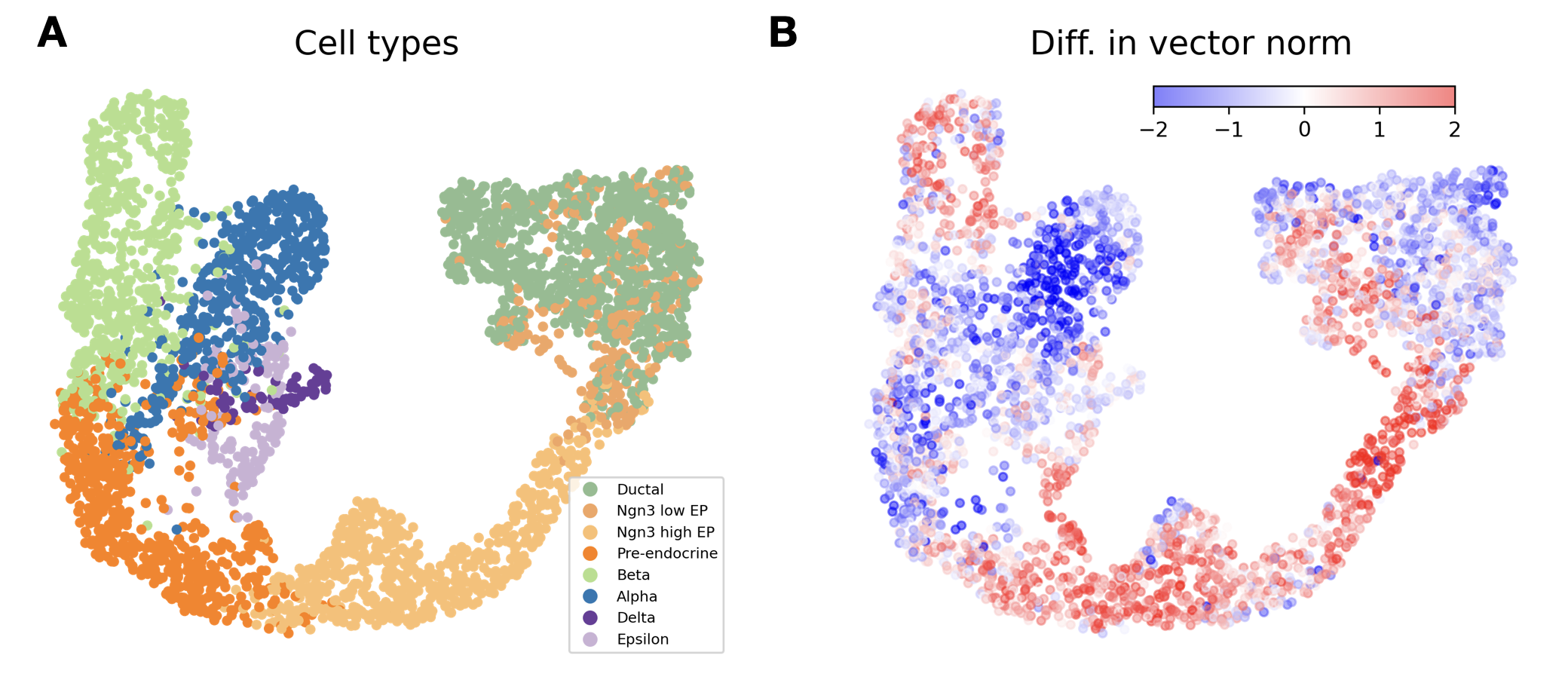

Supplement: S17 Fig — We compare scVelo velocities projected by scVelo v1 to κ-velo velocities projected by Nyström-projection v2 for every cell. (A) UMAP colored by cell types. (B) Difference in the norm of the two vectors ‖v1‖ − ‖v2‖. (TIFF) [file pcbi.1010031.s019.tiff]

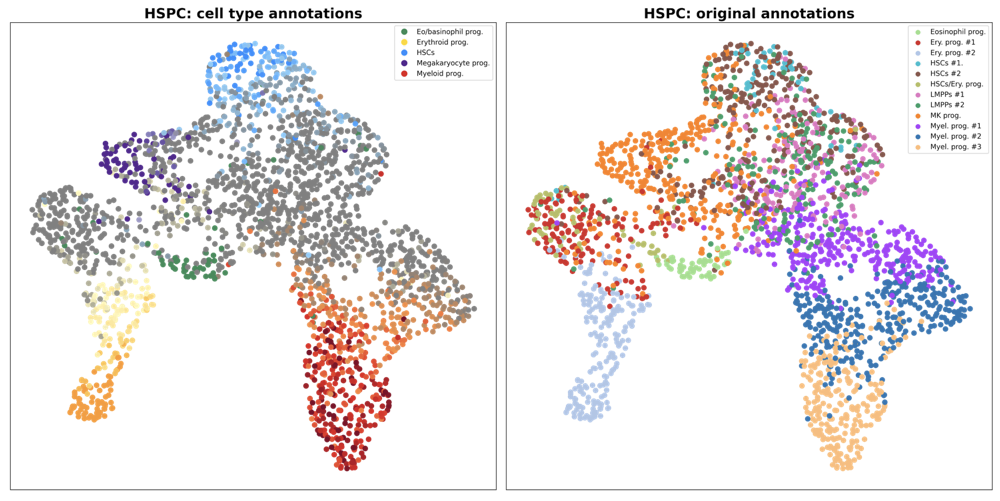

Supplement: S18 Fig — Cells are coloured for (A) our assigned cell types (see Note I in S1 Appendix) or (B) the cell types assignments from the original data analysis [23]. (TIFF) [file pcbi.1010031.s020.tiff]

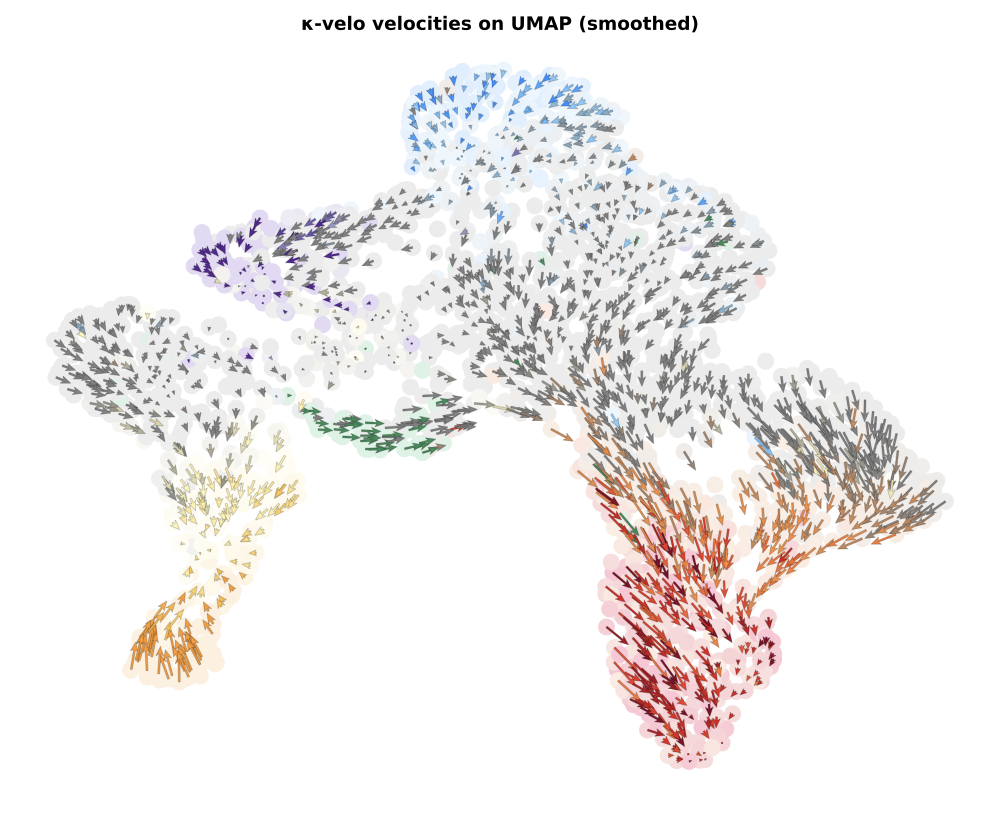

Supplement: S19 Fig — Velocities were smoothed by averaging over the 30 nearest neighbours. Neighbourhoods are calculated in S space. Non-smoothed projection in main Fig 5B. (TIFF) [file pcbi.1010031.s021.tiff]

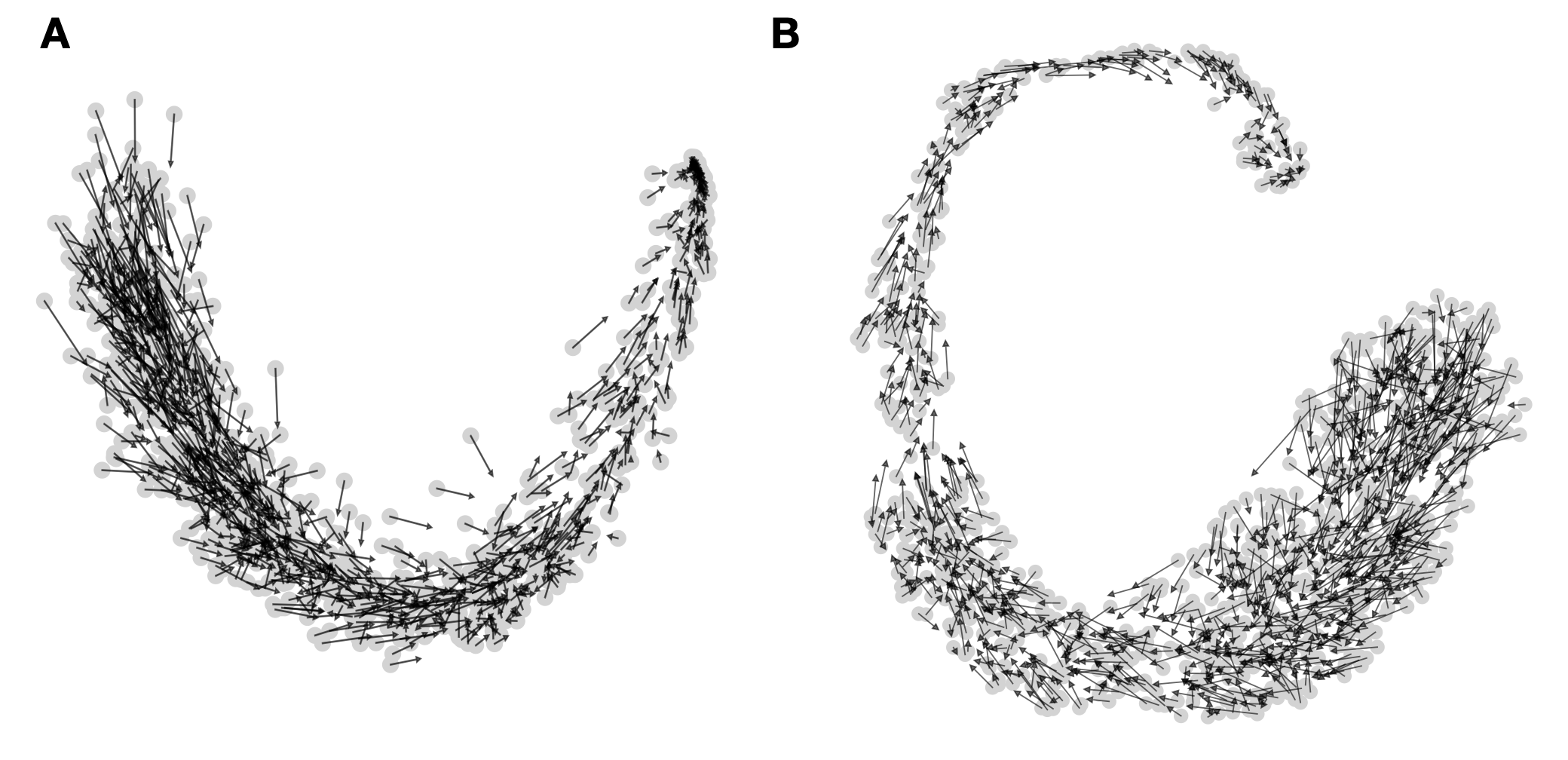

Supplement: S20 Fig — (TIFF) [file pcbi.1010031.s022.tiff]

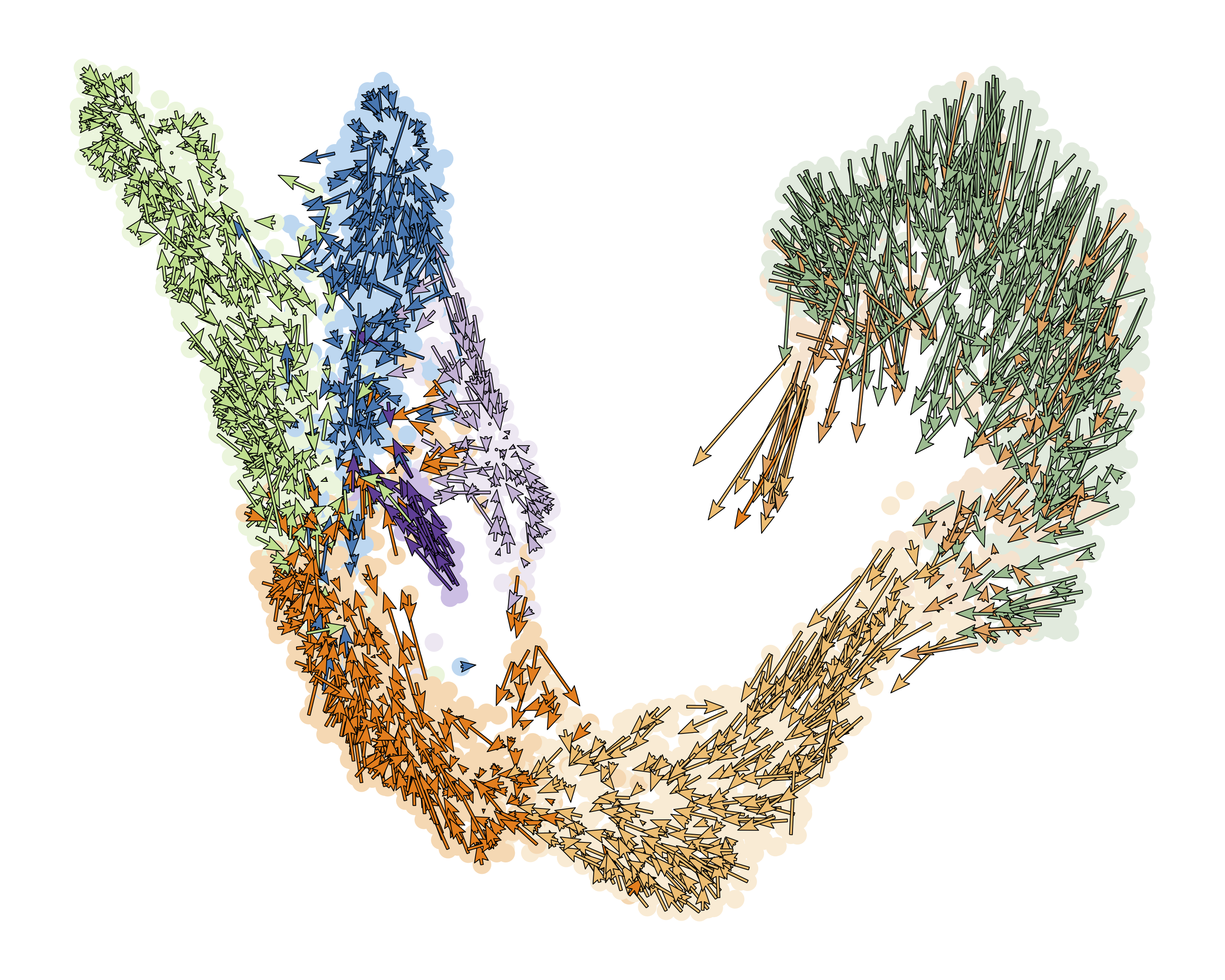

Supplement: S21 Fig — Velocities were smoothed by averaging over the 50 nearest neighbours. Neighbourhoods are calculated in S space. (TIFF) [file pcbi.1010031.s023.tiff]

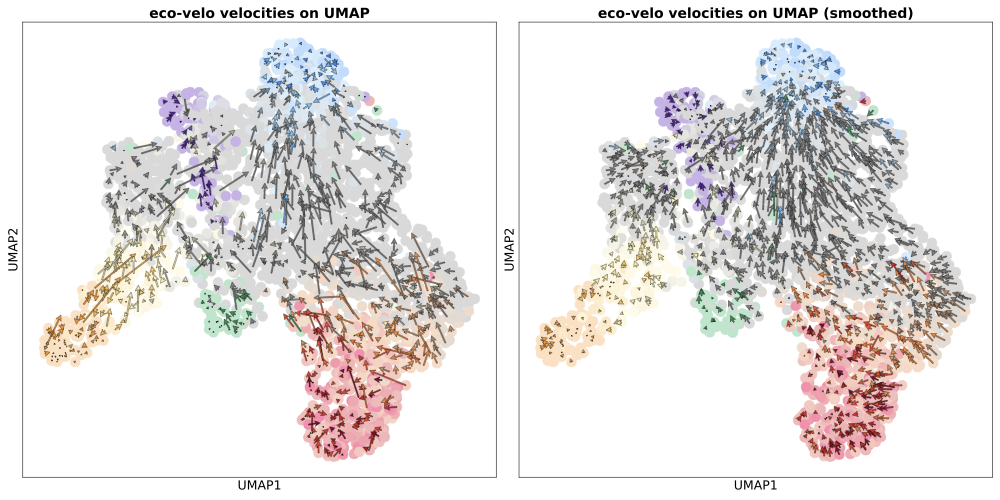

Supplement: S22 Fig — Left: raw vector visualisation, right: smoothed vector visualisation. Like scVelo (main Fig 5C), the velocities point from the more differentiated populations back to the stem cells. (TIFF) [file pcbi.1010031.s024.tiff]

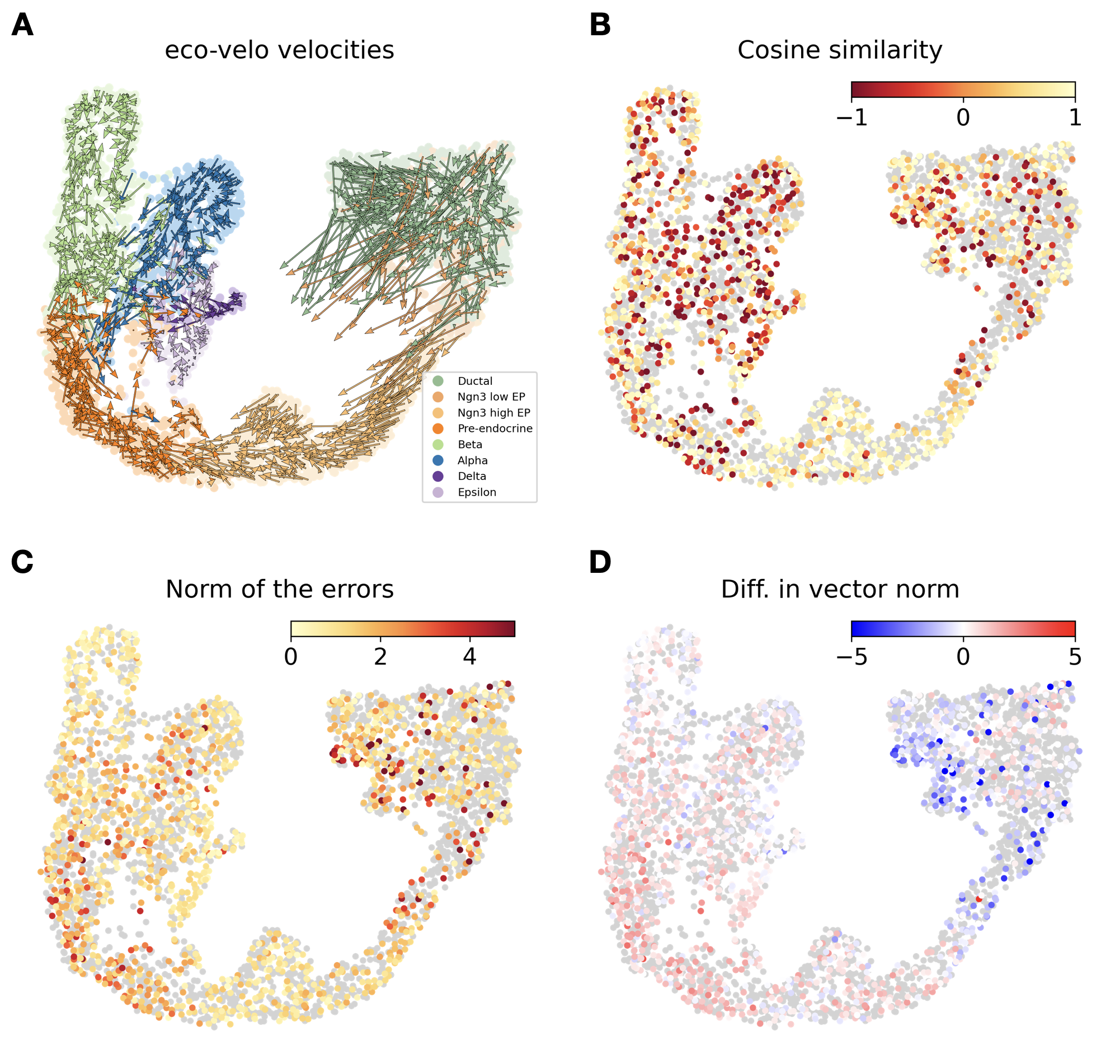

Supplement: S23 Fig — We compare κ-velo velocities projected by Nyström-projection v1 to eco-velo velocities projected onto the UMAP calculated in the κ-velo pipeline and shown in main Fig 4 for every cell. (A) UMAP colored by cell types. (B) Cosine similarity between the two vectors. (C) Norm of the difference between the two vectors ‖v→1-v→2‖. (D) Difference in the norm of the two vectors ‖v→1‖-‖v→2‖. Cells are colored in grey when we do not have a velocity value for eco-velo, i.e. the cell does not have a mutual nearest neighbour within the top 50 neighbours. (TIFF) [file pcbi.1010031.s025.tiff]
