# Supplementary material for: Towards reliable quantification of cell state velocities
Source: PLoS Comput Biol. 2022 Sep 28;18(9):e1010031. doi: 10.1371/journal.pcbi.1010031 (PMC9550177; doi:10.1371/journal.pcbi.1010031)
Supplement: Attachment — Submitted filename: review.pdf [file pcbi.1010031.s027.pdf]

Review of *'Towards reliable quantification of cell state velocities'*

May 2022

1 Summary

This article develops k-velo: a new method for analyzing RNA velocity, and a computationally-efficient approximation: eco-velo.

Figure 3 shows results on a simulated dataset. The method appears to perform significantly better than scVelo. Figure 4 shows results on a real scRNA-seq dataset.

Overall, the article seems less thorough than the recent work "RNA-velocity unraveled". This work is briefly mentioned and it would be nice to compare to it more fully and understand the relationship between this work and that work.

2 Comments

Related to Figure 3D,E: The data are projected onto 2 principal components in order to compare velocity vectors. Is this to reduce effects of noise? How do the results look with different numbers of principal components?

For the second plot in Fig 3E: Given that the vectors are normalized to have length 1, it is not clear how the vectors could have different lengths. What is being plotted in Fig 3E?

Fig 3E: Can the authors show a histogram of the norm of the errors? Fig 4: Comparing D and E, there appear to be massive differences in the different visualization techniques and also the methods (C,D). It would be nice to do a more quantitative comparison of the velocities produced by k-velo and scVelo. For example, one could make a 2d scatter plot with a point for each cell showing the differences in magnitude (x-axis) and direction (y-axis) between the two methods. This could also be done for the simulated dataset, where the ground truth is known. Coloring the cells by the norm of the error (as requested above, although previously I requested a histogram) could reveal if the methods agree on cells where both methods have low error (two colorings are possible, one for the error of each method).

A quantitative comparison of k-velo and eco-velo could be done, similar to figure 3. If k-velo is faster than scVelo, when would one use eco-velo?

3 Typos

There is a "kappa" outside of math mode on page 5.
