# Supplementary material for: Towards reliable quantification of cell state velocities
Source: PLoS Comput Biol. 2022 Sep 28;18(9):e1010031. doi: 10.1371/journal.pcbi.1010031 (PMC9550177; doi:10.1371/journal.pcbi.1010031)
Supplement: Attachment — Submitted filename: Response_First_Revision.pdf [file pcbi.1010031.s028.pdf]

Response to the Reviewers

Title: Towards reliable quantification of cell state velocities

Manuscript Reference Number:
PCOMPBIOL-D-22-00426

Authors:
Valérie Marot-Lassauzaie
Brigitte Joanne Bouman
Fearghal Declan Donaghy
Yasmin Demerdash
Marieke Alida Gertruda Essers
Laleh Haghverdi

Date: Friday July 22, 2022

Dear Editor and Reviewers,

Thank you very much for taking the time to assist us with this manuscript. We believe the reviewers' comments have vastly helped us improve the clarity and quality of our manuscript. In the revised version, we have addressed all points raised by the expert reviewers to the best of our knowledge. All major changes to the manuscript are marked in red font. Updated figures are outlined in red. In the response file, we mark the quotations from the manuscript text in *Italic*. We summarise the major changes to the manuscript below.

We have revised the methods description and writing of the text extensively to improve clarity and relation with other parallel studies as well as addressing other enquires of the reviewers including the premises of eco-velo's development.

We fully agreed with the reviewers that the method needed to be tested on more datasets. We have added the results of applying our pipeline to a single-cell dataset of hematopoietic stem and progenitor cells (HSPCs), which is a system for which both velocyto and scVelo predict overall velocities reverse to the known pseudotime order of cell type clusters. Additionally, we have included a dataset produced by smart-seq2 sequencing technology with subpopulations of Schwann cell precursors (SCPs) differentiating into chromaffin cells.

To make the methods accessible for use, we have now wrapped up the functions in a python package that allows users to run the entire κ-velo and eco-velo pipeline from spliced / unspliced count matrices. To allow compatibility with Scanpy and scVelo the pipeline works with AnnData objects. We have also added some Jupyter Notebook tutorials to show and explain the typical analysis workflow.

In the updated package, we have made three changes to the κ-velo pipeline. Previously, we used scVelo parameter recovery implementation to infer the reaction rate parameters α, β and γ. However, we had some critics to their approach, and to resolve these issues we have now implemented our own parameter recovery function. Second, we now use another interpretation of the Nyström projection method which does not require matrix inversion, as that could be (at least analytically) a burden when the transition matrix is not full rank. Third, in application to the newly included datasets, we noticed issues with fitting of genes that do not display evident kinetics on the u-s phase portraits. Given prior information on the pseudotemporal order of cell clusters, we now filter genes with fitted state assignments that conflict with this prior information. We have modified the corresponding description of each method part in the main text as well.

Sincerely,
Valérie Marot-Lassauzaie,
Brigitte Joanne Bouman,
Fearghal Declan Donaghy,
Yasmin Demerdash,
Marieke Alida Gertruda Essers,
Laleh Haghverdi

1 Response to Reviewer #1

The authors present two approaches, κ-velo and eco-velo, one detailed and one heuristic, to address current challenges in single-cell RNA velocity inference. κ-velo tackles the scaling invariance issue of rate parameters in existing velocity estimation methods by approximating time steps with cell densities. Eco-velo utilizes mutual nearest neighbors between unspliced and spliced expression matrices to rapidly derive a transition map among cells. The authors also developed a new visualization method that can nonlinearly embed velocity vectors into low-dimensional representations using Nystrom projection. Overall, the manuscript is well written and easy to read. The authors show improved velocity fields and parameter stability in both simulated and real datasets. However, there are a few important issues to address and/or clarify before one can conclude the validity and applicability of the methods.

We thank the reviewer for their positive evaluation as well as helpful suggestions and feedback. In the revised manuscript we improve the clarity of our motivations for the novelties and contributions we make in this work. We also implement a few modifications to the method as suggested by the reviewer.

1.1 Justification of problem formulation: The authors state that there is a scaling invariance issue in the current velocity ODE formulation and that scVelo circumvents this issue by assuming a constant prior time of 20 hrs for all genes. However, it seems that the solution of $(\kappa\alpha, \kappa\beta, \kappa\gamma)$ would lead to the same expression levels of U and S in the analytical solutions iff the continuous time-vector t is also scaled down by the same factor of κ, as t/κ. The scaling invariance issue becomes less obvious under the assumption that each cell can be assigned to a single globally shared time-point like proposed in scVelo. More clarification on the issue would be helpful, perhaps stating what alternative assumptions are used and citing any previous studies referred to in section 2.1.2 ("It has been shown that there is a scaling invariance...").

We thank the reviewer for their comment. In our updated manuscript section 2.1.2, we address equivalence of the parameters scaling with that of time as well as the relation between a global latent time with the scale invariance more clearly. We fully agree that inference of a global (shared across all genes) latent time for each cell would resolve the scale invariance issue of the gene-wise ODEs. However, assignment of the global latent time is not part of the EM and parameter inference in scVelo (the term "prior information" used in the scVelo manuscript, mainly refers to the absolute time of the process in hours and not to the usage of a global latent time as part of the optimisation procedure). Rather, a separate latent time is inferred for cells for each gene separately. Then the cells' global latent time is assigned using a heuristic voting approach considering the latent time from multiple genes. This approach still keeps scVelo's initial assumption of the same constant full cycle for all genes and fails to assign different time scales for fast versus slow changing genes.

Given a global latent time assignment of the cells that is close to the true time (e.g. the density scaled pseudotime, or the pseudotime order of cells), we could also use it to recover the scaling factors with our κ-velo approach. We added Fig. S8 to demonstrate on simulation that using the true global latent time of the cells resolves the scale-invariance and now refer to this figure in section 3.2. Alternatively we could use other proxies for this global latent time s.a. the pseudotemporal order of cells, or a density corrected version of it called universal time [1]. However, this would require separating several differentiation branches and calculation of pseudotime time along each branch, which would introduce new types of errors and uncertainties associated with trajectory

inference and branch assignment. To avoid these issues, we choose to approximate true time using density per gene in κ-velo.

As we rewrote and restructured section 2.1.2 extensively to address this question, we kindly ask the reviewer to check this section in our revised manuscript.

1.2 Latent time alternative:

By no longer applying the shared cell time assumption, it seems that the newly proposed methods lose the ability to recover a latent time scale. In the interest of finding a global developmental pseudotime as one of the characteristics of each cell that is useful for many downstream analyses, perhaps the authors can derive an alternative approach to rank cells on a shared time axis.

Recovery of a global differentiation latent time based on velocities would be a very useful feature. However, even beyond the fact that scale invariance issues were not addressed properly previously (i.e., by assumption of constant time for all genes), we have some critics to the current approach for the recovery of the global latent time in scVelo. First, the gene-specific time assignment for each gene: it helps the procedure of fitting of the reaction rate parameters in scVelo, but unless cells go into upregulation at the start of the process and immediately go into the phase of downregulation after reaching the upregulations' peak, it does not reflect the true time of a cell's progression through the dynamical process. That is because we do not know the time that genes spend in steady-state. Take the example of a process happening over a course of 20 hours, and one gene that is activated at half time of the process (=10 hours) for which we want to assign times to each cells. Cells on the beginning of the phase portrait will be assigned a small time of e.g. 1 hour, when the true time would be closer to 11 hours. Such cases are very common in the processes we looked at. One example is given in the pancreas dataset by the glucagon gene *Gcg* shown in Fig. 4B that is only upregulated in the alpha cells.

Second, we also do not fully agree with the integration of this assigned time over all genes. scVelo assigns a latent time to each cell, but as this time is not the same for the same cell across parameter fitting on different genes, the method then uses a "voting" approach to integrate these estimates across genes after inferring roots. scVelo argues that the optimal value is found at the 20-30% quantiles. If we have a cell with an assigned time of (1, 10, 3, 12) across four genes, does that mean that the true time is 3? Can we assume that the errors in these assignments simply cancel out?

In fact in κ-velo, we also assign a (temporal) order to the cells when fitting the parameters for each gene. We save these estimates in the same format as scVelo and therefore the user is in principle able to recover the gene-wise latent time, and use scVelo's voting procedure to assign a global latent time to each cell. However, because of aforementioned concerns, we believe recovery of a global latent time based on velocities needs to be done more carefully in follow-up studies e.g. with strategies similar to CellRank [2], where several individual cells' state transitions are chained together to construct longer transition paths. This would allow for recovery of a global latent time which can be more accurate than classical geometrically recovered pseudotime, e.g. in packages such as diffusion pseudotime [1]. We have added a paragraph in the discussion to address this:

"The recovered high-dimensional velocity vectors now contain the deterministic part, but also capture the stochasticity of the dynamics. This can be used to perform several downstream analyses and answer questions about cell's progress through the dynamical process. In the past, recovery of cell specific global latent time has been done after velocity analysis [3]. The recovery of a cell's global time was based on a heuristic integration of time assignment from individual genes. However, the gene-wise assignment of latent time are error-prone and additionally do not take into account the

time that genes spend in steady-state. Integrating these errors does not necessarily mean that they cancel out. Because of these two reasons, recovery of global latent time should be done more carefully in follow up studies with strategies similar to CellRank [2], where several sequential cell state transitions are chained together to construct long transition paths along the differentiation manifold. Alternatively, estimation of global latent time may be integrated in the expectation maximisation procedure, similar to the approach in a recent preprint [4]."

1.3 Cell density as a proxy for Δt: It remains to be seen how accurate/reliable it is to approximate Δt or equivalently, the inverse of velocity with cell density. The authors state that "In snapshot data, the probability of capturing cells in a specific region of the expression space is proportional to the time cells typically spend in that region." Usually, only total RNA is considered. Because unspliced and spliced counts are separately examined in RNA velocity, and their respective velocity patterns in the same transient states may be different (Fig. 1C, La Manno et al. 2018 [5]), it is worth testing the density method for both U and S. The concern is that unspliced-based time inversion formula in equation (6) may not be accurate enough to be applied to spliced-based velocity $V = dS/dt$.

One way to test this is to compute the correlation of the inferred velocities from κ-velo or velocities from scVelo with cell densities, computed from either U or S.

In addition, it would be interesting to see if this method is applicable to different sequencing techniques/platforms, for example, Smart-seq2 or single-nucleus RNA-seq.

Thank you for your comment. We have tested the kappa inference method both for U and S and we find the values to be correlated (Figure S8). Note that we do the kappa estimation on the u counts after the initial processing steps (as described in our pipeline) which include smoothing based on neighborhood defined by the spliced counts of the cells. Therefore, inaccuracy of the u counts is less of a problem for density estimation. Since the equation for Δt from u depends on two estimated parameters (α, β), while the equation for Δt from s also depends on an additional estimated parameter γ, we preferred to use the equation of Δt from u. In down-regulation $(\alpha = 0)$, the equation of Δt from u depends on only a single parameter. We address this point in the methods section 2.1.3:

"Here, we inferred κ from the unspliced counts data. One could similarly use the spliced counts data and infer κ from the $s(t)$ solution in Equation 5, which yields κ estimations congruent with those inferred from the unspliced data (see Supplementary note 4 and Fig. S3). However, as $u(t)$ depends only on α, β while $s(t)$ depends on α, β, γ, i.e. on one more imputed parameter, we consider recovery of κ values from $u(t)$ as more straightforward and less error-prone."

Furthermore, in our updated package, the function to recover κ from density now has the parameter "mode", which the user can set to decide whether kappa should be estimated from u (default) or from s.

Additionally, we shared the reviewers interest in seeing our algorithms perform on different sequencing techniques. Unfortunately, recovering RNA velocities for single-nucleus datasets remains a challenge in the field. In their review on future challenges in RNA velocity, Bergen et al. discuss the problems associated with single-nucleus experiments in more detail [?]. In short, the relative abundances of spliced and unspliced mRNAs are different in the nucleus only, compared to the whole single cell. In other words, the ratio of spliced to unspliced counts might be different from gene to gene depending when we measure in the nucleus only. To use single-nucleus datasets we would need to know how the relative abundances are different and factor the differences into the mathematical formulations.

It is true that we expect different sequencing technologies to give us different data quality. Technologies sequencing the whole transcript length will likely capture more introns than 3' biased technologies. That is simply because few genes may have intronic regions within 70-100bp of the 3'end. Technologies with UMI will also return more accurate counts than technologies without UMIs, where we might count the same transcript multiple times. We agree that in future work it would be interesting to assess how the differences in the technologies could affect the input (raw) U and S matrices. However, in this work we start with the raw U and S matrices and therefore we beleive that such an analysis is beyond the scope of this work. To validate the methods on a different technology than 10X, we also analysed a SMART-seq2 dataset. This dataset covers subpopulations of Schwann cell precursors (SCPs) differentiating into chromaffin cells [5], for which we describe the results in Figure S5.

1.4 The similar β and γ assumption:

Eco-velo assumes similar spliced and degradation rates for all genes. This needs more confirmation. Under the steady-state model, this would mean that the steady-state expressions of u and s are equal for all genes. The Velocyto example of circadian cycle genes doesn't directly support this claim but does provide justification for an alternative MNN-based approach. It would be helpful to show some example genes that do possess such rates.

At steady state we have:

$$\frac{du}{dt} = \alpha - \beta u = 0 \qquad \Leftrightarrow u_\infty = \frac{\alpha}{\beta}$$

$$\frac{ds}{dt} = \beta u - \gamma s = 0 \qquad \Leftrightarrow s_\infty = u_\infty \frac{\beta}{\gamma} \Leftrightarrow s_\infty = \frac{\alpha}{\gamma}$$

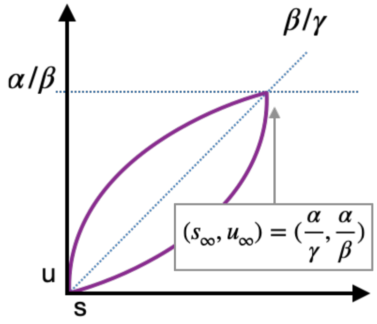

Expression values at steady-state.

Therefore, in the steady state solution given by the intersection of the horizontal and the diagonal blue lines in the figure to the right, both u_∞ and s_∞ depend on the transcription rate α which can be different for different genes. In Equation 11 and 12 (eco-velo) we describe how having $s(t+\Delta t) \propto u(t)$, implies $\Delta t = 1/\gamma$ for the genes thus similar γ across the observed genes. Now, if β was different across genes, the proportionality still holds but the with a different coefficient β/γ for each gene. That's why for eco-velo (which does not decompose the high-dimensional velocity vectors into gene-wise components) we ideally would select genes with similar γ as well as β. We now include an additional paragraph in the section 2.1.4 (Methods) to make this clearer:

"The strong assumptions of eco-velo (similar γ and β across genes) may not hold for every biological processes and every subset of genes. Thus here, one would ideally select a set of genes that are only transcriptionally regulated (via α), but not post-transcriptionally regulated (involving gene-specific β and γ rates). An example of such a cases seems to occur in Fig. 1e of the original RNA velocity paper [La Manno 2018], where the authors observed for bulk RNA-seq measurements of cell cycle genes in the mouse liver over a time course of the circadian cycle, that unspliced mRNAs appear predictive of spliced mRNA at the next time point with a similar signal intensity coefficient. Conditioned on its assumptions, eco-velo (in contrast to the methods based on gene-wise parameter estimation) specifies the time interval of the reported velocities and also skips several error-prone parameter estimation and data smoothing steps. How much the different assumptions of each method are satisfied for different experimental settings, data qualities, as well as the purposes

of velocity analysis (e.g. estimating the overall velocity directions or obtaining the average cell state velocities over a specific time scale) would determine which method is more appropriate to use."

In snapshot data we do not know the true rates of genes. However, an example of a set of genes studied in time-course data with similar β and γ is given by the circadian cycle genes studied in the velocyto paper (see Figure below). In the case of the pancreas dataset, we expect the cell cycle genes to be on a different time scale than other genes, and therefore, we now exclude them from the eco-velo analysis.

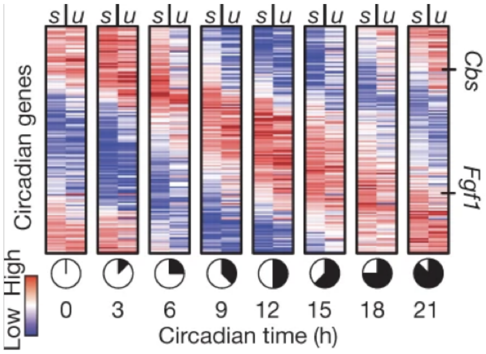

Fig. 1e of the velocyto paper [5] Abundance of spliced (s) and unspliced (u) mRNAs for circadian-associated genes in the mouse liver over a 24-h time course12. The unspliced mRNAs appear as predictive of spliced mRNA at the next time point, with a similar signal intensity coefficient.

1.5 The authors claim that noisy velocity vectors around root cells represent high plasticity and capture more biological variances, and thus are preferable to a smoothed and consistent velocity flow. It is hard to see this with data generated from a single branch. Maybe a more comprehensive simulation (for example using dyngen) such as bifurcating or loop-based lineages can showcase this idea more clearly.

To fully answer this comment, we will clarify the motivation behind the simulation example shown in figure 2. Our goal was to show how different visualisation methods (1) capture regions with both high- or low variance and speed and (2) how the methods could handle projecting new states to a space outside of the space observed in the original set, which arises if the trajectory is cut off before reaching a stationary cell type, and the velocities point to future states not yet observed in the data. To show both of these aspects, we generated a simulation with high variance in root cell states, and faster velocities and small variance towards the end of the branch. We do not aim to claim that root cell states are necessarily associated with larger variance, or that the end of the shown trajectory represents true terminal states. In fact, if we let the same simulation run for longer time, the velocities would go down again and we would reach a second region of high variance. An example of a process similar to the simulation example would be illustrated by the pancreas endocrinogenesis dataset if the cells had been sampled at an earlier time point, where the Ngn3 cells (in orange on the UMAP) have left the cycle but not yet committed to the terminal fates.

Because our goal was to show these two characteristics of the visualisation, we believe that a more complex simulation (with branching) would not make this aspect more clear. A loop-based lineage would also not illustrate the second aspect. However, we now recognise that the reasoning behind the simulation was not well described in the work and extended the description of the simulation. The text in Section 3.1. now reads:

"Ideally, a visualisation of cell state velocities should faithfully represent all aspects of the high-dimensional vector. The visualisation should respect the direction of velocity vectors as well as their magnitude (speed of change). This can be particularly difficult if the new states are in gene space not yet observed in the original set, e.g. the velocities point further than existing points. The embedding should also preserve local variations, representing fluctuations of the dynamics and cell plasticities. To assess these points, we compare existing RNA velocity visualisation methods with ours, on simulated data where the true high-dimensional velocities are known and do not need to be inferred. We design a simulation to assess all these aspects of the projection. In that simulation the cells follow a hidden true time with a high variance at the beginning and faster transitions towards the end of the trajectory (Section 2.5). The final stable cell state is not yet reached in our simulation, and the velocities of the latest cells point towards not yet observed future states.".

1.6 Both κ-velo and eco-velo need to be tested on more real datasets other than the pancreatic example. The full mouse gastrulation dataset is potentially usable. Others include the dentate gyrus neurogenesis dataset in the scVelo paper [3].

We fully agree that we have to show our algorithms on different datasets. Besides the dataset of SCPs differentiating into chromaffin cells that we added, we also decided to include a dataset of murine HSPCs. The reason that we choose to include this particular dataset is because there have been reports of RNA velocity algorithms being unable to recover the correct vectors in HSPC datasets [6, **?**]. Indeed, we show in figure 5 that we are able to correctly recover the velocity vectors, in contrast to the previous pipelines is scVelo which incorrectly yielded velocities directing from more differentiated cells to more stem-like cells.

We were unfortunately unable to use the complete gastrulation dataset, as it is in fact a mixture of multiple time points and batches of measurement. Instead we apply κ-velo on one of the batches from this data set (Figure S13). Several batch correction algorithms exist, but little is known about the effects of these algorithms on the two connected modalities of unspliced and spliced counts. Batch correcting datasets for RNA velocity has been described as one of the future challenges in the field of RNA velocity by Bergen et al. [**?**], and we also refer to it now in the discussion section:

"Estimation of cell state velocities in presence of multiple time point measurements or multiple batches of data collection is another important problem. However, the solution is not trivial as existing batch effect correction methods can distort the proportions between the s and u counts from separate batches. One possible strategy can be to estimate the velocities within each batch separately and visualise and project the estimated velocities on a shared embedding of all batches. Investigation of different approaches and possibilities remain open."

1.7 κ-velo uses scVelo's state assignment result. However, because scVelo fits each gene separately, conflicting/wrong transient state assignments and trajectory directions can arise among genes with high likelihoods (also mentioned in Fig. 4B). A filtering step to detect such wrongly assigned genes may improve the result.

We thank the reviewer for their suggestion and include it in a revised version of the method. We agree that conflicting state assignments still arise in high-likelihood genes, and that this could be circumvented by leveraging information across genes. During this work, we also noticed a few further problems with scVelo's parameter estimation implementation which altogether motivated us to revise it and write our own curve fitting and parameter estimation procedure. The problems with scVelo's procedure include:

- assignment of latent time: by assigning latent time to the cells in scVelo's EM step the parameters often reach a local minima. We factor out the time from the equation of u(t) and fit s(u) instead, which removes the need to fit a latent time as an independent parameter. We explain this time-independent u-s curve fitting approach in detail in section 2.1.3 of the manuscript.
- scVelo rescales U and S separately in the fitting step s.t. they have the same variance. As explained in our Note S1 this is equivalent to setting $\beta \approx \gamma$, and does not allow a full fitting of reaction rate parameters. Unfortunately the consequences of this step is not transparent in the scVelo paper. Instead, if needed we allow to fit a scaling parameter instead of setting it to a fixed value and explain the motivation behind this in note S1, as well as making this step transparent in the main text section 2.1.3, Equation 7.

We have also taken the suggestion of reviewer 1 to heart and implemented an additional filtering step to the κ-velo pipeline. After removing genes with low likelihoods, we include another filtering step to remove genes where the state assignment conflicts with prior knowledge of the cluster ordering. The details of the filtering step are described in section 3.3 where we write:

"Even after filtering of low-likelihood genes, we still find genes where the recovered dynamics do not match the known order of cell types. For example, early upregulation or late downregulation can often not be easily differentiated based on the u-s phase portrait alone (Fig. S4: step 6). This could ultimately lead to incorrect velocity assignments. To avoid this issue, we can use prior information about the temporal order of cell types to perform one more round of filtering if that information is given (see Note S7: step 6). We use this information to exclude genes where the fitted state assignments of up- or downregulation do not fit the expected state assignments. After both filtering steps, we calculate the low-dimensional embedding on the reduced gene set, so that the embedding only represents space that can be reached by velocities."

1.8 Why are the U and S matrices in eco-velo preprocessing L2 normalized instead of L1 like for k-velo?

In fact, different normalisation methods may be appropriate for different data analyses as also suggested in a few recent publications, including [?]. For eco-velo the first step of the procedure is identification of the MNN pairs between the spliced and unspliced data sets, which requires separate normalisation within each dataset [7]. Weheras L1 and L2 normalisation are very similar concepts (the former is projection on unit cube and the latter is projection on unit sphere), we stick with the L2 normalisation proposal of the original method, motivated by the fact that after L2 normalisation Euclidean and cosine distance between cells become equivalent hence removing a level of arbitrary choice of the metric use. κ-velo, however is not based on any type of distance measurement between the cells, but rather u-s interdependence and dynamics. That is why use of L2 was not particularly preferred over the more widely used L1 (size factor) normalisation.

1.9 What would the result of eco-velo look like if using the standard scVelo preprocessing steps or k-velo preprocessing steps? If it only theoretically makes sense to follow eco-velo's steps, please explain.

As mentioned above, the theory and foundations between the two methods are different and we needed to adapt the processing steps accordingly.

In κ-velo, because the fitted ODEs are based on the relation of u to s we normalise the two matrices together. A separate normalisation would distort the ratio of u to s per cell. In fact, we

show that these issues in normalisation are partially responsible for the artefacts that we see in the MURK genes (Note S7 - step 3, Figure S13). On the other hand eco-velo is based on cell-cell distances between these two modalities. Because of this, we need to treat U and S as separate datasets and thus normalise them separately.

1.10 In the κ-velo pancreas result (Fig. 4C), the velocities are very large near Ductal (root) cells and small towards terminal cell types (Alpha, Beta, etc). This seems to conflict with the simulated data (Fig. 2A). One may expect that a cell's differentiation potential gradually drops as it matures towards a steady cell type. Is it possible that the simulation assumption is reversed? More explanation would be helpful, and any references to existing experimental support would be great.

Thank you for your comment. We tried to explain the motivation for our simulation design with the changes to the main text as described under your comment 1.5. We agree that the differentiation potential should drop at the steady cell types, but in the simulation these final states are not yet reached. With this we wanted to illustrate the difficulty with projecting points onto space not yet seen in the dataset used to create the embedding. This can arise in an experimental setting where sampling of the cells happens before cells maturation in time. Ideally we would like then to be able to determine that the stationary cell state has not been reached yet. We now make it clear in the results interpretation that it is only the linear (PCA) projection approach which has this capability among other projection methods. The text in Section 3.1. now reads:

"We note that, unlike PCA projection, neither scVelo's nor Nyström projection are able to project end of velocity arrows that are out of distribution of existing data points."

1.11 When computing embeddings, what are the meanings of training sets and test sets (Fig. 2C)? Perhaps stating them explicitly as the tails and heads of velocity arrows in Fig. 2 caption would help readers understand.

The Nyström approach is used to project any set of new points onto an existing embedding. With this background, we called the states used to build the original embedding i.e., the s counts as the starting points of the velocity vectors, the "training set", and the states projected to the embedding, i.e., end of the velocity vectors, the "test set". We modified Figure 2C to make this notation clear using the terms "start point of velocity vector" and "end point of velocity vector".

2 Response to Reviewer #2

In this manuscript, the authors aimed to solve several key questions regarding the velocity estimation and visualization. They developed κ-velo which enables the estimation of the relative magnitude of velocity components across genes. At the same time, they developed a new method to visualize the velocity. Using both simulated and real data, the authors demonstrated that their method outperforms scVelo, one of the state-of-the-art methods for velocity estimation and visualization. The main comments and concerns are as follows:

We would like to thank the reviewer for their comprehensive feedback and comments. Your comments have considerably helped us with improving the clarity and application of our methods in the revised manuscript and code.

2.1 The algorithm developed in this manuscript is not available, at least not user-friendly. Scripts in GitHub can only be used to generate figures for this manuscript. It is difficult for users to use this algorithm to analyze their internal data or other public data other than those mentioned in this manuscript.

We fully agree with the reviewers and have now wrapped up the code for this algorithm in a python package that is available on GitHub. The package allows users to run the entire κ-velo and eco-velo pipeline from spliced / unspliced counts matrices. To allow compatibility with Scanpy and scVelo the pipeline works with AnnData objects. We have also added some Jupyter Notebook tutorials to show and explain the typical analysis workflow.

2.2 In the manuscript, the authors address several key issues of velocity estimation and visualisation in the Introduction section. All work in the manuscript is aimed at addressing these issues. Although these issues have been reported in detail in other studies, a brief description of these issues can help readers better understand this manuscript.

Point well taken. We now more completely describe the problem setting and the existing challenges on the scale invariance of velocity components, time intervals of velocity estimation and visualisation problems in the manuscript. In addition to the overview in the introduction, we discuss the existing issues in more detail in the Methods section. We devoted the subsections "2.1.1 Time scale over which average cell state velocities are reported"and "2.1.2 Scale invariance of gene-wise velocity components" to theoretically motivate why new approaches are needed. We now add a new paragraph in the main text. We have also add Figure S8 to show that having the true time (or other forms of a correctly inferred global latent time) would solve the scale invariance issue. Similar to addressing the related question 1.1 of reviewer 1, we kindly ask the reviewer to check the entire section 2.1.2 of our revised manuscript.

For current visualisation issues, in addition to the revised demonstrations of the existing challenges on simulated data in Figure 2, we now modified the methods section 2.2 to theoretically describe the existing problems more clearly:

"A vector summation as proposed in Equation 13 used in velocyto and scVelo is questionable for three reasons. First, this approach is not faithful to the velocity vectors length, e.g., two velocity vectors with the same direction, but different length (in the same neighbourhood) in the high-dimensional space will be visualised with similar lengths. That is because they will be assigned the same P_{ij} as Pearson correlation does not respect the length of the vectors. Second, $\frac{\overrightarrow{Y_j - Y_i}}{\|Y_j - Y_i\|}$ does not in general provide an orthonormal basis as the direction of several neighbouring cells to cell i

can be correlated on the low dimensional embedding. As a result, this approach may change the direction of the velocity vectors depending on how much the orthonormality principle is disturbed for a given neighbourhood. For example, if the chosen neighbourhood extends longer along the differentiation path than its width, velocities will be visualised as more smooth vectors along the path. Third, $(P_{ij} - \frac{1}{n})$ *can be negative even if the velocity direction* $\vec{v_i}$ *is correlated with the direction of a neighbouring cell* j*, which is not correct."*

2.3 In Section 3.1, the authors demonstrate that their PCA embedding method can reliably represent high plasticity at the beginning and commit fate faster at the end of the trajectory through simulated data. However, with the nonlinear projection method, it appears that these phenomena cannot be observed. Does this mean that PCA-based methods and nonlinear projection methods can only show limited aspects of the velocity?

That is correct, on the one hand PCA (and subsequent linear projection of the velocities which would present the ground truth), cannot capture the structural complexity of most datasets. On the other hand, nonlinear projection methods, including the Nystrom projection, cannot capture velocity vectors directing to future states outside of the existing data manifold. We have made this more clear in the revised version of Figure 2 as well as section 2.2.1. which now reads:

"In light of the above, linear projection, e.g. by PCA (Note S5) although not capable to capture the complexity of several datasets which consist of multiple branching events and subpopulations, remains the only approach in which the visualised arrows are a true representation of the high-dimensional velocity vectors. None of the non-parametric nonlinear projection approaches can deal with projection of out of distribution data points, implying that near the boundaries of the differentiation paths, where actual velocities may point to directions going out of the existing manifold of the start point of velocity vectors, velocity visualisations will be less reliable. Moreover, embedding methods which may not keep the continuity of the data manifold (e.g. t-SNE and UMAP) are more prone to the artefacts of out of distribution data points projection."

Moreover, t-SNE or UMAP generally tend to map regions of higher density (i.e. "slow"regions) to a relatively larger space in the embedding than given by the Euclidean distance of cells in the gene space. We can see this in Figure 2: the early states take up a much larger space of the t-sne embedding proportionally to the later states than they do in PCA. As a result the velocity arrows will look proportionally longer in that region, simply because the region is more spread out by the reference embedding. To more clearly accentuate this point, we have added the blue and red outline highlighting the same regions of low and high variance to the t-SNE embedding (Figure 2E) as also shown on PCA (Figure 2A,B,D). We further show how the Nyström projection method on PCA in Fig. 2D and diffusion maps in Fig. S7 captures the velocities. We have also added a paragraph in the text addressing this:

"t-SNE tends to map regions of higher density, e.g. of slower velocities, in gene space to a larger space in the embedding as highlighted by the cells outlined in blue and red. Consequently, the velocity arrows are also visualised in a scale proportional to the distance of cells in a given region on the embedding, hence looking longer than their true length in gene space. On embeddings that do not distort cell to cell distances in the gene space such as PCA or diffusion maps with a constant kernel width, the length of the velocity arrows are well represented by Nyström projection (Fig. S7 diffusion map and Fig. 2D PCA). We note that, unlike PCA projection, neither scVelo's nor Nyström projection are able to project end of velocity arrows that are out of distribution of existing data points."

2.4 The operation of κ-velo does not take much computing resources and time. In this case, is eco-velo mode still necessary? Does this model have any other advantages or might be suitable for specific situations?

We thank the reviewer for pointing out this unclarity, which we now elaborate more on in the main text. One main advantage of eco-velo is that it skips several cumbersome gene-wise parameter-fitting procedures by inferring velocities using displacements for each cell directly in the high-dimensional genes space. Thus, this is not a matter of computation speed only, but also bypassing several sources of error and uncertainty that may be introduced thereby.

Another advantage of eco-velo is that it fixes the time scale over which velocities are reported. This is important because average velocities measured over different time scales can be different, especially for non-smooth dynamics. None of the other existing methods (including κ-velo) are able to handle this issue.

We believe that the different assumptions supporting each method can be more (or less) valid for a given data set and for a specific purpose. One can imagine that fixing the time according to eco-velo is more suitable for comparing RNA-velocities from a given set of genes to velocities inferred from another with a different β and γ, or other data modalities, e.g. mRNA-protein inferred velocities. We now elaborate on these points in the main text:

"Our second approach eco-velo estimates cell state velocities directly in the high-dimensional gene space by calculating the displacement for each cell in a fixed time interval. This approach eliminates the need for cumbersome and error-prone gene-wise parameter estimations. It also specifies the time interval over which high-dimensional velocities are reported, a feature that the gene-wise parameter estimation approaches (including κ-velo) are missing. Specification of the velocity estimation time interval can be important for data sets that include multiple non-smooth-dynamics genes where short-term cell velocities can deviate significantly from their long-term velocity directions."

As well as at the end of that section where we write:

"The strong assumptions of eco-velo (similar γ and β across genes) may not hold for every biological processes and every subset of genes. Thus here, one would ideally select a set of genes that are only transcriptionally regulated (via α), but not post-transcriptionally regulated (involving gene-specific β and γ rates). An example of such a cases seems to occur in Fig. 1e of the original RNA velocity paper [5], where the authors observed for bulk RNA-seq measurements of cell cycle genes in the mouse liver over a time course of the circadian cycle, that unspliced mRNAs appear predictive of spliced mRNA at the next time point with a similar signal intensity coefficient. Conditioned on its assumptions, eco-velo (in contrast to the methods based on gene-wise parameter estimation) specifies the time interval of the reported velocities and also skips several error-prone parameter estimation and data smoothing steps. How much the different assumptions of each method are satisfied for different experimental settings, data qualities, as well as the purposes of velocity analysis (e.g. estimating the overall velocity directions or obtaining the average cell state velocities over a specific time scale) would determine which method is more appropriate to use."

2.5 Are there any other datasets or cell types that can be used to test the performance of κ-velo? For example, single-cell data of different T-cell states.

Consistent with our answer to question 1.6 from reviewer 1, we agree on the importance of checking the performance of our algorithm on other datasets. Therefore, we included a velocity analysis on a dataset of hematopoeietic stem and progenitor cells (HSPCs). We choose this dataset, because of the reported issues with recovering RNA velocity in HSPC datasets [6, **?**]. Additionally, we applied

κ-velo and eco-velo to a dataset of Schwann cell precursors (SCPs) differentiating into chromaffin cells [5]. This dataset was included to test our method on a Smart-Seq2 dataset.

3 Response to Reviewer #3

This article develops κ-velo: a new method for analyzing RNA velocity, and a computationally-efficient approximation: eco-velo. Figure 3 shows results on a simulated dataset. The method appears to perform significantly better than scVelo. Figure 4 shows results on a real scRNA-seq dataset.

We thank the reviewer for their helpful feedback and suggestions, which we believe resulted in improvement of the clarity and quality of our quantitative assessment procedures.

3.1 Overall, the article seems less thorough than the recent work "RNA-velocity unraveled". This work is briefly mentioned and it would be nice to compare to it more fully and understand the relationship between this work and that work.

Our manuscript is a parallel and independent study that we have submitted back-to-back with the "RNA-velocity unraveled" work to PLOS computational biology. To keep the independence of our study and findings, we abstain from direct comparison and integration of opinions from the two manuscripts. However, as a hint to the methodological differences between the two studies, we have modified parts of the main text (Introduction) as follows:

"In parallel to this study, [8] and [9] point out some of the limitations and problems of current velocity visualisation methods. [8] also suggests that, due to the highly stochastic nature of gene expression process, currently used (deterministic) approaches are insufficient and propose development of probabilistic alternatives. More recently, a variational inference method for RNA velocity estimation has also become available [4]."

"In this manuscript, we argue that when dealing with highly stochastic processes, deterministic approaches are only useful when talking about average velocities over specific time intervals, instead of talking about spontaneous velocities which are immeasurable in real life. We propose two different approaches for estimation and visualisation of RNA velocities. In κ-velo, we first design a processing workflow specifically adapted to downstream velocity calculations, thereby addressing problems in previously used workflows. We then solve for the gene-wise reaction rate parameters and propose an approach to relate velocity components across genes, hence resolving the scale invariance issue. We also present a new visualisation method that more faithfully represents the stochastic part of the velocities. In addition, we propose eco-velo, a heuristic method that bypasses several cumbersome, computationally costly and stochasticity killing steps used by other available methods."

The parallel study "RNA-velocity unraveled" does share similar opinions on some of the existing problems addressed in our work including visualisation, (pre)processing and parameter estimation, however mostly focuses on different aspects of the analysis than we do. For example, for (pre)processing, Gorin et al. focus on the assignment of spliced and unspliced reads, whereas we focus on later processing steps and investigate the s-u normalisation and upscaling of u. We show that correct preprocessing resolves issues that were previously reported as problematic for parameter fitting (MURK genes).

Gorin et al. suggest a fundamentally different (probabilistic) approach for parameter estimation suggesting it could describe the dynamics of the system more precisely than the currently used deterministic ODEs. However, their propositions remain suggestive and descriptive without testing improved methods or algorithms. They also do not discuss the scale invariance issues that arises when combining velocity components from multiple genes.

We, on the other hand, propose new algorithms and demonstrate their performance on improving the analysis for simulation and real data. We adjust the processing pipeline, and resolve the

scale invariance with κ-velo. We also suggest the adaptation of the Nyström projection theorem for visualisation of velocities. In eco-velo we fix the time scale over which (average) velocities are reported in the deterministic frameworks, a feature that is not accounted for in other existing approaches, but is important when describing the highly stochastic dynamics of mRNA expression and splicing by deterministic ODEs.

3.2 Related to Figure 3D,E: The data are projected onto 2 principal components in order to compare velocity vectors. Is this to reduce effects of noise? How do the results look with different numbers of principal components?

Thank you for your feedback on the clarity of Figure 3, which we have modified now. There are multiple aspects of the recovered velocity vector that we want to assess to understand the performance of the different methods. We tried to have a look at all these aspects in this figure, and maybe packed it too densely.

In Figure 3D, we look at the differences in the high-dimensional velocity vector. If we were to plot this for only a single gene, the points would be distributed around a line. The interesting aspect here is the comparison of multiple genes, the components of which will be combined to make the high-D velocity vectors. We can see that the velocities as returned by scVelo are "overdispersed" in comparison to κ-velo. In fact, this is proportional to the difference in speed of genes (e.g. to the scaling factor κ). To make this aspect more clear, we have replaced Figure 3D with a figure in which we only compare the velocities for two genes of different speed. The previous comparison of all genes has been moved to the supplement Fig. S9A.

Because the high-dimensional vector is not directly conceivable to the human mind, low-dimensional representations of the velocities are usually used for interpretation of the result. That is why we want to assess the changes on lower-dimensional representation in the visualisation returned by the algorithm. However, we now also include the high-dimensional version of Fig. 3E in the supplement, Fig. S9B. In section 2.2. and Fig.2, we argue that PCA is the only representation where the projected velocities are a true representation of the high-dimensional velocity vector. Since multiple genes contribute to each PC, we expect the angels distribution to be different in PCA compared to high-D space. In this relatively simple simulation the main axis of variance is well captured by the two first PCs. To better show this, we included the PCA embedding in the supplement Fig S10, and explain it in the main text Section 3.2.:

"Because the high-dimensional vector is not directly conceivable to the human mind, low-dimensional representations of the velocities are usually used for interpretation of the result. We also compare the vectors after projection on a PCA embedding (Fig. S10) and find that they are also much closer to truth, both for direction and length (Fig. 3E and S11). Here, for both κ-velo and scVelo, we find the biggest errors in regions of lowest and highest velocities, but scVelo's errors are much higher than κ-velo's."

3.3 For the second plot in Fig 3E: Given that the vectors are normalized to have length 1, it is not clear how the vectors could have different lengths. What is being plotted in Fig 3E?

We note that normalising the velocity norms across the cells is just a multiplication of all high-dimensional velocities by the same constant, hence keeping differences of velocity length across different cells. In this figure, we want to compare the recovered velocities to a known ground truth. However, each methods to recover the velocities returns results that are on very different scales, making them difficult to directly compare. We normalise the variance of the high-dimensional

velocity vector, but do not change the mean. To make this more clear, we have now changed the legend of the figure to read: *"The high-dimensional velocity vectors are normalised to have equal variance for ease of comparison."*. Note that the vectors can still have different lengths after this normalisation, and in particular they can still have different lengths on the low-dimensional embedding as different genes contribute differently to the different PCs.

3.4 Fig 3E: Can the authors show a histogram of the norm of the errors?

Figure 3E showed the cosine similarity to measure whether the vectors point in the correct direction and the difference in the norms of the vectors $\|\vec{v_1}\| - \|\vec{v_2}\|$ to measure if the magnitude of the vectors are different. It is true that these two aspects are also brought together in the norm of the errors $\|\vec{v_1} - \vec{v_2}\|$, which we have also included in supplement Fig. S11A.

3.5 Fig 4: Comparing D and E, there appear to be massive differences in the different visualization techniques and also the methods (C,D). It would be nice to do a more quantitative comparison of the velocities produced by k-velo and scVelo. For example, one could make a 2d scatter plot with a point for each cell showing the differences in magnitude (x-axis) and direction (y-axis) between the two methods. This could also be done for the simulated dataset, where the ground truth is known. Coloring the cells by the norm of the error (as requested above, although previously I requested a histogram) could reveal if the methods agree on cells where both methods have low error (two colorings are possible, one for the error of each method).

We thank the reviewer for the suggestion, and have added plots to partly enable comparison between velocities from different methods. A burden for quantitative comparisons on real data is that the gene space of the high-dimensional scVelo and κ-velo vectors are different. That is because for κ-velo we subset to only high u- and s- counts genes, as well as high-likelihood genes which scVelo does not do. We can however do this comparison on simulation, which we now add to the revised manuscript (S10D-E). This quantitative comparison allows us to see that the biggest differences between prediction and truth are found in regions of highest and lowest velocities for both methods, although the errors of scVelo are larger than those of κ-velo. We add this to Section 3.2. where we explain Figure 3E as well:

> *"Because the high-dimensional vector is not directly conceivable to the human mind, low-dimensional representations of the velocities are usually used for interpretation of the result. We also compare the vectors after projection on a PCA embedding (Fig. S10) and find that they are also much closer to truth, both for direction and length (Fig. 3E and S11). Here, for both κ-velo and scVelo, we find the biggest errors in regions of lowest and highest velocities, but scVelo's errors are much higher than κ-velo's."*

While direct comparison on real data is difficult due to the difference in selected gene sets, we can still compare the results for the velocities projected on non-linear embedding. We now include this for the pancreas endocrinogenesis dataset in main figure 4F-G and supplementary figure S17, to allow readers to more easily asses the regions where the methods disagree the most. We see the highest disagreement in direction (Cosine similarity ≈ -1) in the cycling ductal population, as well as in the end of the branches of alpha, beta, epsilon and delta cells. In particular the direction in the small population of delta cells is wrongly recovered by scVelo. The highest errors are found in the alpha and Ngn3 high endocrine progenitor cells as the two methods predict different magnitudes of velocities in these regions. We also now refer to these subfigures in the manuscript:

"Comparing the projected velocities of the full scVelo pipeline (Fig. 4E) to κ-velo pipeline (Fig. 4C), we see that the methods most strongly disagree in the high-plasticity ductal population (Fig. 4F-G, S17). There is also a strong disagreement in the delta cells, which scVelo predicts to differentiate into the alpha cells, as well as in the alpha cells themselves that are predicted to have very small velocities all along the branch. Looking at single genes u-s phase portrait such as the Gcg gene (Fig. 4B), we see that the cells are still differentiating and the full alpha branch has not reached the terminal state yet.".

3.6 A quantitative comparison of k-velo and eco-velo could be done, similar to figure 3.

Here too, it is not completely correct to directly compare eco-velo and κ-velo as the velocities are calculated using different sets of genes thus visualised on different data embeddings, whereas it is more correct to use the same set of genes for making the UMAPs and visualisation as those that have been used for velocity inference. To still get an overall feeling of the conflict between these methods we use eco-velo to project the velocitites onto the UMAP of κ-velo now shown in supplementary figure S23. These plots highlight the high variance of eco-velo's result and the agreement between the embedded velocitites for the two methods can vary greatly between neighbouring cells. This variance could come from errors in the selection of MNNs (which could be due to gene sets that do not match the assumptions) or it could represent true variance in cell states. At the moment, it is not possible to determine the true source of this variation. We also describe this comparison in the main text:

"A quantitative comparison of the projected velocities from eco-velo and κ-velo is shown in Fig. S23, where we see a strong similarity in the Ngn3 low endocrine progenitor, but more variation between the methods in the cycling ductal population as well as in the terminal states."

3.7 If k-velo is faster than scVelo, when would one use eco-velo?

We thank the reviewer for their comment. In fact, the other reviewers raised similar questions which prompted us to clarify the advantages of eco-velo conditioned on validity of its assumptions. Please see our response to question 1.4 and 2.4. in the current document as well as the revised section 2.1.4 on eco-velo in the main text.

3.8 There is a "kappa" outside of math mode on page 5.

Corrected, thank you for catching this!

References

[1] Haghverdi L, Büttner M, Wolf FA, Buettner F, Theis FJ. Diffusion pseudotime robustly reconstructs lineage branching. Nature methods. 2016;13(10):845-8.

[2] Lange M, Bergen V, Klein M, Setty M, Reuter B, Bakhti M, et al. CellRank for directed single-cell fate mapping. Nature methods. 2022:1-12.

[3] Bergen V, Lange M, Peidli S, Wolf FA, Theis FJ. Generalizing RNA velocity to transient cell states through dynamical modeling. Nature Biotechnology. 2020 Dec;38(12):1408-14.

[4] Gu Y, Blaauw DT, Welch J. Variational Mixtures of ODEs for Inferring Cellular Gene Expression Dynamics. In: International Conference on Machine Learning. PMLR; 2022. p. 7887-901.

[5] La Manno G, Soldatov R, Zeisel A, Braun E, Hochgerner H, Petukhov V, et al. RNA velocity of single cells. Nature. 2018 Aug;560(7719):494-8.

[6] Barile M, Imaz-Rosshandler I, Inzani I, Ghazanfar S, Nichols J, Marioni JC, et al. Coordinated changes in gene expression kinetics underlie both mouse and human erythroid maturation. Genome Biol. 2021 07;22(1):197.

[7] Haghverdi L, Lun AT, Morgan MD, Marioni JC. Batch effects in single-cell RNA-sequencing data are corrected by matching mutual nearest neighbors. Nature biotechnology. 2018;36(5):421-7.

[8] Gorin G, Fang M, Chari T, Pachter L. RNA velocity unraveled. bioRxiv. 2022.

[9] Zheng SC, Stein-O'Brien G, Boukas L, Goff LA, Hansen KD. Pumping the brakes on RNA velocity–understanding and interpreting RNA velocity estimates. bioRxiv. 2022.
