# Supplementary material for: Towards reliable quantification of cell state velocities
Source: PLoS Comput Biol. 2022 Sep 28;18(9):e1010031. doi: 10.1371/journal.pcbi.1010031 (PMC9550177; doi:10.1371/journal.pcbi.1010031)
Supplement: Attachment — Submitted filename: PLOS-towards-reliable.docx [file pcbi.1010031.s026.docx]

Review of Towards reliable quantification of cell state velocities

The authors present two approaches, κ-velo and eco-velo, one detailed and one heuristic, to address current challenges in single-cell RNA velocity inference. Κ-velo tackles the scaling invariance issue of rate parameters in existing velocity estimation methods by approximating time steps with cell densities. Eco-velo utilizes mutual nearest neighbors between unspliced and spliced expression matrices to rapidly derive a transition map among cells. The authors also developed a new visualization method that can nonlinearly embed velocity vectors into low-dimensional representations using Nystrom projection. Overall, the manuscript is well written and easy to read. The authors show improved velocity fields and parameter stability in both simulated and real datasets. However, there are a few important issues to address and/or clarify before one can conclude the validity and applicability of the methods.

Comments:

Major

1. Justification of problem formulation:

The authors state that there is a scaling invariance issue in the current velocity ODE formulation and that scVelo circumvents this issue by assuming a constant prior time of 20 hrs for all genes. However, it seems that the solution of (kα, kβ, kγ) would lead to the same expression levels of U and S in the analytical solutions iff the continuous time-vector t is also scaled down by the same factor of k, as t / k. The scaling invariance issue becomes less obvious under the assumption that each cell can be assigned to a single globally shared time-point like proposed in scVelo. More clarification on the issue would be helpful, perhaps stating what alternative assumptions are used and citing any previous studies referred to in section 2.1.2 (“It has been shown that there is a scaling invariance…”).

2. Latent time alternative:

By no long applying the shared cell time assumption, it seems that the newly proposed methods lose the ability to recover a latent time scale. In the interest of finding a global developmental pseudotime as one of the characteristics of each cell that is useful for many downstream analyses, perhaps the authors can derive an alternative approach to rank cells on a shared time axis.

3. Cell density as a proxy for Δt:

It remains to be seen how accurate/reliable it is to approximate Δt or equivalently, the inverse of velocity with cell density. The authors state that “In snapshot data, the probability of capturing cells in a specific region of the expression space is proportional to the time cells typically spend in that region.” Usually, only total RNA is considered. Because unspliced and spliced counts are separately examined in RNA velocity, and their respective velocity patterns in the same transient states may be different (Fig. 1C, La Manno et al. 2018), it is worth testing the density method for both U and S. The concern is that unspliced-based time inversion formula in equation (6) may not be accurate enough to be applied to spliced-based velocity V = dS/dt.

One way to test this is to compute the correlation of the inferred velocities from k-velo or velocities from scVelo with cell densities, computed from either U or S.

In addition, it would be interesting to see if this method is applicable to different sequencing techniques/platforms, for example, Smart-seq2 or single-nucleus RNA-seq.

4. The similar β and γ assumption:

Eco-velo assumes similar spliced and degradation rates for all genes. This needs more confirmation. Under the steady-state model, this would mean that the steady-state expressions of u and s are equal for all genes. The Velocyto example of circadian cycle genes doesn’t directly support this claim but does provide justification for an alternative MNN-based approach. It would be helpful to show some example genes that do possess such rates.

5. The authors claim that noisy velocity vectors around root cells represent high plasticity and capture more biological variances, and thus are preferable to a smoothed and consistent velocity flow. It is hard to see this with data generated from a single branch. Maybe a more comprehensive simulation (for example using dyngen) such as bifurcating or loop-based lineages can showcase this idea more clearly.
6. Both k-velo and eco-velo need to be tested on more real datasets other than the pancreatic example. The full mouse gastrulation dataset is potentially usable. Others include the dentate gyrus neurogenesis dataset in the scVelo paper (Bergen et al. 2020).

Minor

1. K-velo uses scVelo’s state assignment result. However, because scVelo fits each gene separately, conflicting/wrong transient state assignments and trajectory directions can arise among genes with high likelihoods (also mentioned in Fig. 4B). A filtering step to detect such wrongly assigned genes may improve the result.
2. Why are the U and S matrices in eco-velo preprocessing L2 normalized instead of L1 like for k-velo?
3. What would the result of eco-velo look like if using the standard scVelo preprocessing steps or k-velo preprocessing steps? If it only theoretically makes sense to follow eco-velo’s steps, please explain.
4. In the k-velo pancreas result (Fig. 4C), the velocities are very large near Ductal (root) cells and small towards terminal cell types (Alpha, Beta, etc). This seems to conflict with the simulated data (Fig. 2A). One may expect that a cell’s differentiation potential gradually drops as it matures towards a steady cell type. Is it possible that the simulation assumption is reversed? More explanation would be helpful, and any references to existing experimental support would be great.
5. When computing embeddings, what are the meanings of training sets and test sets (Fig. 2C)? Perhaps stating them explicitly as the tails and heads of velocity arrows in Fig. 2 caption would help readers understand.
